# Updating algal evolutionary relationships through plastid genome sequencing: did alveolate plastids emerge through endosymbiosis of an ochrophyte?

**DOI:** 10.1038/srep10134

**Published:** 2015-05-28

**Authors:** Tereza Ševčíková, Aleš Horák, Vladimír Klimeš, Veronika Zbránková, Elif Demir-Hilton, Sebastian Sudek, Jerry Jenkins, Jeremy Schmutz, Pavel Přibyl, Jan Fousek, Čestmír Vlček, B. Franz Lang, Miroslav Oborník, Alexandra Z. Worden, Marek Eliáš

**Affiliations:** 1 University of Ostrava, Faculty of Science, Department of Biology and Ecology, Life Science Research Centre, Chittussiho 10, 710 00 Ostrava, Czech Republic; 2 Institute of Parasitology, Biology Centre, Czech Academy of Sciences, Branišovská 31, 370 05 České Budějovice, Czech Republic; 3 University of South Bohemia, Faculty of Science, Branišovská 1760, 370 05 České Budějovice, Czech Republic; 4 Monterey Bay Aquarium Research Institute (MBARI), Moss Landing, CA 95039, USA; 5 US Department of Energy Joint Genome Institute, Walnut Creek, California 94598, USA; 6 HudsonAlpha Institute for Biotechnology, 601 Genome Way NW, Huntsville, Alabama 35806, USA; 7 Centre for Algology and Biorefinery Research Centre of Competence, Institute of Botany, Czech Academy of Sciences, Dukelská 135, 379 82 Třeboň, Czech Republic; 8 Institute of Molecular Genetics, Czech Academy of Sciences, Vídeňská 1083, 142 20 Prague 4, Czech Republic; 9 Département de Biochimie, Centre Robert-Cedergren, Université de Montréal, 2900 Boulevard Edouard Montpetit, Montréal, Québec, H3C 3J7, Canada; 10 Integrated Microbial Biodiversity Program, Canadian Institute for Advanced Research, Toronto, M5G 1Z8, Canada

## Abstract

Algae with secondary plastids of a red algal origin, such as ochrophytes (photosynthetic stramenopiles), are diverse and ecologically important, yet their evolutionary history remains controversial. We sequenced plastid genomes of two ochrophytes, *Ochromonas* sp. CCMP1393 (Chrysophyceae) and *Trachydiscus minutus* (Eustigmatophyceae). A shared split of the *clpC* gene as well as phylogenomic analyses of concatenated protein sequences demonstrated that chrysophytes and eustigmatophytes form a clade, the Limnista, exhibiting an unexpectedly elevated rate of plastid gene evolution. Our analyses also indicate that the root of the ochrophyte phylogeny falls between the recently redefined Khakista and Phaeista assemblages. Taking advantage of the expanded sampling of plastid genome sequences, we revisited the phylogenetic position of the plastid of *Vitrella brassicaformis*, a member of Alveolata with the least derived plastid genome known for the whole group. The results varied depending on the dataset and phylogenetic method employed, but suggested that the *Vitrella* plastids emerged from a deep ochrophyte lineage rather than being derived vertically from a hypothetical plastid-bearing common ancestor of alveolates and stramenopiles. Thus, we hypothesize that the plastid in *Vitrella*, and potentially in other alveolates, may have been acquired by an endosymbiosis of an early ochrophyte.

The evolutionary history of photosynthetic eukaryotes is astonishingly complex. One of the most puzzling aspects is the evolution of plastids derived from red algae (rhodophytes) by eukaryote-to-eukaryote endosymbioses^1^,^2^. Phylogenetic analyses and a number of shared features indicate a single origin of these plastids, implying a scenario in which an early red alga became integrated into a heterotrophic eukaryotic host cell and ultimately gave rise to diverse algal lineages as well as non-photosynthetic lineages that subsequently lost their plastids. However, how the distribution of red alga-derived plastids was achieved across these various lineages remains controversial.

The Ochrophyta, a monophyletic phylum within the Stramenopiles (or Heterokonta), are the most diverse algal group with secondary plastids of algal origin in terms of morphology, pigmentation and phylogeny^3^,^4^. Diatoms (Bacillariophyceae) and multicellular brown algae (Phaeophyceae) are the best characterized ochrophytes, but at least 15 separate “classes”, plus several isolated smaller lineages of uncertain taxonomic status exist^4^,^5^,^6^,^7^,^8^. Several groups within the ochrophytes have important ecological roles, particularly in terms of marine photosynthesis and uptake of CO_2_^9^.

Understanding relationships among the ochrophytes is important for both ecological and evolutionary studies. Phylogenetic studies of nuclear SSU rRNA gene sequences proposed the existence of several higher-order clades, specifically: diatoms plus the Bolidophyceae; the Chrysophyceae plus the Synchromophyceae; and the large “PX” clade comprising brown algae, the Xanthophyceae and other less well-known groups^4^,^5^,^6^,^7^,^8^. Multi-gene datasets including various combinations of nuclear, plastid and mitochondrial genes have improved resolution of ochrophyte phylogenetic relationships, yet some relationships are still unresolved^5^,^7^,^10^. Most notably, the position of the root of the ochrophyte phylogeny remains unknown. Despite these uncertainties and sometimes lack of statistical support, many different putative groupings of ochrophyte classes have been proposed^11^. This includes the Limnista, which contain the classes Eustigmatophyceae and Chrysophyceae along with a few minor lineages.

Regardless occasional horizontal movement of plastids between different eukaryotic lineages (see below), it is generally assumed that within most algal and plant lineages plastids are inherited vertically. The growing list of completely sequenced plastid genomes of various algal and plant lineages is thus a resource for inferring relationships among both plastids and host cell lineages^12^,^13^,^14^. Given the lack of evidence for non-vertical inheritance of ochrophyte plastids, plastid genome sequences could be also very helpful in resolving the ochrophyte phylogeny, but only seven of the approximately 15 known ochrophyte classes are represented by completely sequenced plastid genomes (Table S1). Furthermore, sampling is limited to a single genus in the case of eustigmatophytes, and to a single species in the raphidophytes and xanthophytes. Representatives of other classes, such as the Chrysophyceae, Pinguiophyceae and Dichtyochophyceae, are yet to be sequenced.

Two different conceptual frameworks are often used to explain the emergence of ochrophytes. One is the “chromalveolate hypothesis”, which posits that all extant groups with red-algal derived plastids, namely the Ochrophyta, Myzozoa (a subgroup of the Alveolata that includes the Apicomplexa, Dinoflagellata, “chromerid” algae and a few additional minor lineages), Haptophyta, and Cryptophyta (a subgroup of the Cryptista along with some non-photosyntetic lineages), inherited their plastids vertically from a common ancestor^15^,^16^. Plastid-lacking lineages closely related to any of these groups (i.e. plastid-lacking stramenopiles, alveolates and cryptists) would have lost the plastid secondarily. However, phylogenetic and phylogenomic analyses have failed to provide evidence for the monophyly of the proposed “chromalveolate” lineages. Whereas stramenopiles and alveolates are specifically related to the Rhizaria^17^,^18^, cryptists and haptophytes do not show any robustly supported affiliation and in some analyses are even found nested among eukaryotes with the primary plastid (Archaeplastida)^18^,^19^,^20^.

The second conceptual framework builds on a growing amount of data suggesting that a red algal plastid was acquired by one “chromalveolate” lineage, from which it spread to others through a series of higher-order tertiary or even quaternary endosymbioses (see, e.g.,^21^,^22^,^23^,^24^,^25^). This scenario has recently been dubbed the “rhodoplex hypothesis”^26^. However, higher-order endosymbiotic gains of plastids postulated by the rhodoplex hypothesis have only been conclusively demonstrated for some dinoflagellate lineages^2^,^27^.

Here, we sequenced and analyzed plastid genomes from two unrepresented ochrophyte groups. *Trachydiscus minutus* CCALA 838 belongs to a newly recognized eustigmatophyte subgroup (Goniochloridales)^28^,^29^ and is deeply diverged from the biotechnologically significant genus *Nannochloropsis*. *Ochromonas* sp. CCMP1393 is a marine member of the Chrysophyceae (Fig. S1), a group long known from freshwater habitats, but recently shown to be widely distributed in marine environments and important for primary production^30^,^31^. We performed comparative and phylogenetic analyses of these genomes in the context of existing plastid sequence data to address questions on relationships among ochrophytes as well as on the plastid evolution in “chromalveolates”.

## Material and methods

### Sequencing and annotation of the plastid genomes

Cultivation of the algae and DNA isolation followed standard protocols. Sequencing of total DNA preparations employed the 454 and Illumina platforms. Initial assemblies of the reads were searched to identify scaffolds corresponding to the plastid genome and the final assembly of the plastid genome sequences was achieved by manual gap filling and polishing. MFannot (http://megasun.bch.umontreal.ca/cgi-bin/mfannot/mfannotInterface.pl) was used for obtaining an initial automated annotation of the assembled plastid genome sequences, which was checked and improved manually. Details on the cultivation, DNA isolation, sequencing, assembly, and annotation are provided in Supplementary methods. Annotated plastid genome sequences from *T. minutus* and *Ochromonas* sp. CCMP1393 are deposited in the GenBank database (accession numbers KJ624065 and KJ877675, respectively).

## Phylogenetic analyses

68 orthologous protein sequences encoded by plastid genomes were aligned and concatenated, leaving 16,948 reliably aligned amino acid (aa) positions in the main (“Full”) alignment (hereafter referred to as dataset F). Removal of the fastest-evolving category of sites from the full dataset F yielded the dataset SP (“Slow Positions”) comprising 11,208 aa positions. The dataset SG (“Slow Genes” comprising 14,699 reliably aligned aa positions was built by aligning only protein sequences encoded by 34 slowly evolving plastid genes defined in a previous study^12^.

Phylogenetic trees were inferred using two different methods: ML (RAxML 7.4.8a)^32^ with the site-homogeneous GTRGAMMA model of amino acid substitution, and Bayesian inference (PhyloBayes 3.3f)^33^ with the site-heterogeneous CAT-GTR model. A detailed description of the procedures used to build the alignments and infer trees is available in Supplementary methods.

## Results and Discussion

### The plastid genomes of *Trachydiscus minutus* and *Ochromonas* sp. CCMP1393 reveal split *clpC* genes and rapid gene evolution shared by eustigmatophytes and chrysophytes

The newly sequenced plastid genomes exhibit size, GC content, and gene content similar to previously sequence plastid genomes (see^1^,^34^; Tables 1 and S2, Fig. S2). Both genomes also display the typical circular-mapping architecture and the presence of inverted repeat (IR) regions separated by a large single copy and a small single copy region. The IR copies are identical at the nucleotide sequence level except for one-nucleotide deletion in one of the *psaC* copies in *Trachydiscus* introducing a frame-shift into the coding sequence; this copy is thus probably a non-functional pseudogene. The IR region of the *Ochromonas* plastid genome is the second longest among ochrophyte plastid genomes sequenced so far, while the small single copy region is exceptionally short (805 bp) and includes only two protein-coding genes.

The set of genes in the plastid genomes of *Trachydiscus* and *Ochromonas* is similar to other ochrophytes in terms of both their number and identity (Tables 1, S2, and S3). The most significant observations concerning the gene complement are discussed below, while additional details are provided in Supplementary Note 1.

One notable feature is the presence of a predicted group I intron in one of the three tRNA-Leu genes in the *Trachydiscus* genome. A tRNA-Leu gene intron has been postulated to have been present in the cyanobacterial ancestor of plastids and in time was lost, presumably independently, in many plastid-containing lineages^35^. Although overlooked in previous publications, we identified a tRNA-Leu gene in *Nannochloropsis* plastid genomes that also contains a group I intron (Fig. S3). This suggests that the intron may be a common feature in eustigmatophytes. In contrast, no introns were identified in *Ochromonas* plastid tRNA-Leu genes. This result is in accord with the previous indication that the intron was lost in the chrysophyte ancestor^35^.

Other interesting features were observed as well. For example, plastid genomes of *Trachydiscus* and *Nannochloropsis* spp. share possession of the gene *ycf49*. This gene codes for small uncharacterized proteins possessing the DUF2499 domain of an unknown function and is also found in primary plastid genomes from cyanidiophyte red algae and the glaucophyte *Cyanophora paradoxa*, but no other published plastid genomes (primary or secondary; Table S4). This is the case even when sensitive PSI-BLAST searches are employed. In both cyanidiophytes and eustigmatophytes *ycf49* genes are located in the same conserved plastid gene block *petL-**ycf49**-ycf4-trnG-psbE-psbF-psbL-psbJ*. Thus, the *ycf49* genes in the eustigmatophyte plastid genomes were apparently inherited directly from the plastid genome of the red algal ancestor of the “chromalveolate” plastids, while most “chromalveolate” lineages lost the *ycf49* gene from their plastid genomes independently on several occasions.

Additionally, results were interesting for the gene *clpC*, which encodes a member of the Clp/Hsp100 family of AAA+ proteins involved in the protein degradation pathway mediated by the ClpP protease. Starkenburg *et al.*^36^ recently demonstrated that in *Nannochloropsis* spp. *clpC* is split into three separate genes, annotated as *clpN* (encoding the N-terminal domain of a typical ClpC protein), *clpC1* (encoding the first AAA ATPase domain), and *clpC2* (encoding the second AAA ATPase domain). We identified these three genes in the *Trachydiscus* plastid genome (with *clpC2* gene present in two identical copies, since it resides in the IR region). This indicates that the split of *clpC* into three genes must have occurred before the radiation of known eustigmatophytes. Furthermore, the *Ochromonas* plastid genome exhibits an intermediate state, as it harbours a separate *clpN* gene, but the rest of the *clpC* gene is intact and codes for both AAA ATPase domains. Our results demonstrate that the split of the 5’-end of the *clpC* gene most likely predates the divergence of eustigmatophytes and chrysophytes, but without a plastid genome from the Pinguiophyceae, a putative sister group of the Limnista^7^, it cannot yet be ascertained whether this split is exclusive (i.e. synapomorphic) for the Limnista.

While inspecting multiple alignments of protein sequences encoded by the plastid genes we also noted that the sequences from eustigmatophytes and/or *Ochromonas* tend to harbour unusual indels in regions otherwise well conserved among ochrophytes (see Fig. S4), which may be an indication that the evolutionary rates of plastid genes in eustigmatophytes and *Ochromonas* are elevated compared to the rates exhibited by other ochrophytes. Indeed, phylogenetic analyses using individual genes revealed noticeably longer branches for eustigmatophyte and *Ochromonas* than for other ochrophytes (Fig. S5), indicating an increased rate of substitutions in the former lineages.

## Phylogenomic analyses of plastid genomes support the Limnista clade

The most recent phylogenomic analyses of algal plastid genomes^12^,^23^,^34^,^37^ did not include data from chrysophytes and eustigmatophytes. Therefore, we conducted analyses using genes encoded by the new plastid genome sequences along with other recently published sequences. We used three different concatenated alignments (F, SG, and SP) and two methods of phylogenetic inference (ML and Bayesian inference). These employed site-homogeneous and site-heterogeneous substitution models to evaluate the robustness of the results (see Material and Methods and Supplementary methods for technical details).

**Figure 1:**
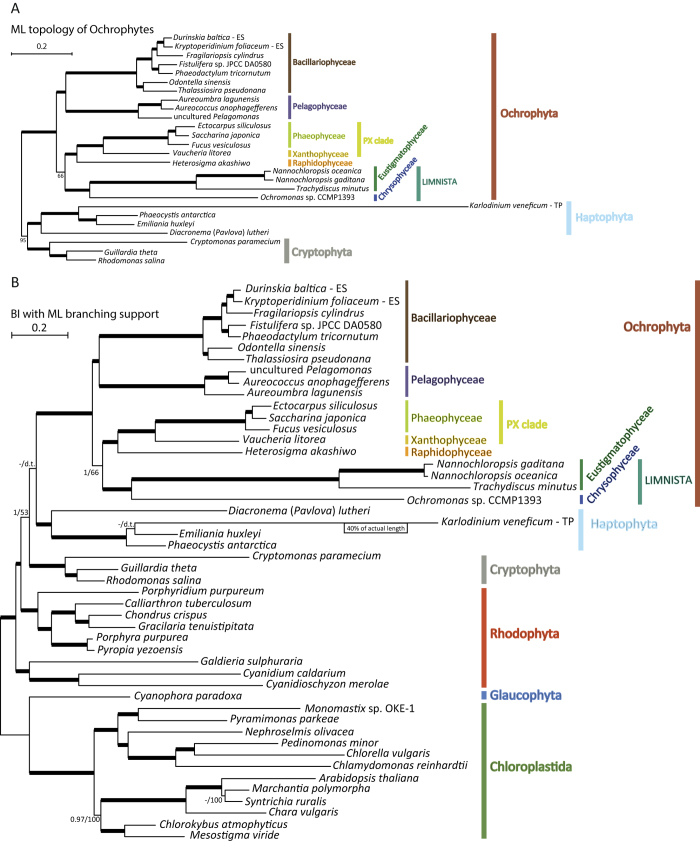
Plastid phylogeny inferred from protein sequences encoded by 34 slowly-evolving conserved plastid genes (dataset SG, 14,699 aa positions). (**A**) Maximum-likelihood tree (RAxML, GTRGAMMA model); only the “chromalveolate” subtree is shown for simplicity. Thick branches received 100% bootstrap support, otherwise the bootstrap support values are indicated by numbers when higher than 50%. (**B**) PhyloBayes tree inferred using the CAT-GTR model. Thick branches were supported by 1.00 posterior probability and 100% bootstrap support values from the ML analyses, otherwise posterior probabilities / bootstrap support values are indicated by numbers when higher than 0.90 / 50%; “d.t.” means that the respective bipartition does not exist in the ML tree.

The six trees obtained from the three different datasets and two methods were generally congruent with the previous analyses and with each other (Fig. 1, S6, and S7, Table 2; informative aspects of our analyses that are not directly related to ochrophytes are provided in Supplementary Note 2). Both ML and Bayesian trees were consistent with a common origin of plastids of all “chromalveolates” from a deep red algal lineage and showed strong to maximal statistical support for the monophyly of ochrophyte plastids. Likewise, haptophyte plastids, including the haptophyte-derived tertiary plastid of the dinoflagellate *Karlodinium veneficum*, were monophyletic, as were those of cryptophytes. Among ochrophyte classes, all those represented by more than one species were monophyletic with maximal support in all trees.

Notably, the chrysophyte *Ochromonas* and eustigmatophytes formed a clade with maximal support in all trees (Fig. 1, S6, and S7). The chrysophyte and eustigmatophyte branches are considerably longer than those of most other taxa included in our analyses, apparently as a result of the generally increased evolutionary rate of individual plastid genes (see above). Such long branches are known to be prone to misplacement due to long-branch attraction, especially when the substitution model employed does not sufficiently capture the actual substitution process^38^,^39^,^40^. However, the clade uniting the *Ochromonas* plastid sequences with those of eustigmatophytes is consistently recovered even with the CAT-GTR substitution model, which is considered effective in coping with long-branch attraction^39^. Together with the unique trait shared by the plastid genome of *Ochromonas* and eustigmatophytes, i.e. the split *clpC* gene (see the previous section), the specific relationship observed between chrysophytes and eustigmatophytes is strongly supported by our analyses.

Our results thus corroborate the existence of the Limnista, a group originally proposed to be comprised of the Chrysophyceae (including Synurophyceae), the Eustigmatophyceae and two enigmatic organisms – the alga *Chlamydomyxa labyrinthuloides* (now known to represent a broader group called the Synchromophyceae^41^) and the minute marine flagellate *Picophagus flagellatus*^11^. While some analyses have supported such a grouping^5^,^7^, others have not^10^. Although our analysis does not include data from the other proposed Limnista lineages (i.e., synchromophytes and *Picophagus*), their affinity to the Chrysophyceae is consistently supported by other phylogenetic analyses^7^,^8^,^41^,^42^. Thus, our results can be interpreted as direct evidence for the monophyly of the Limnista *sensu* Cavalier-Smith and Chao^11^. Whether the Limnista should be expanded to also include the marine class Pinguiophyceae as recently suggested^8^, is a matter for future investigations.

## Identifying the root of the ochrophyte phylogeny

In addition to resolving the Limnista, our analyses provide insights into other parts of the ochrophyte phylogeny. All trees provided maximal support for the sisterhood of brown algae and *Vaucheria litorea* representing the Xanthophyceae. This result agrees with previous single-gene and multi-gene analyses that established the existence of the so-called “PX” clade^5^,^7^. Diatoms and pelagophytes consistently formed sister groups within a single clade, generally with strong support (Table 2). The same clade was seen in plastid phylogenies reported by previous analyses lacking representatives of Limnista^12^,^14^,^34^. This clade likely also includes two additional classes with no sequenced plastid genome to date – the Bolidophyceae and Dictyochophyceae^7^, and was proposed by Riisberg^10^ to be termed the Khakista (expanding the original meaning of this name coined for a grouping of diatoms and bolidophytes only^11^). Our results thus add support to an emerging consensus on the existence of this major ochrophyte subclade united by some potential synapomorphies on the ultrastructural and biochemical level, e.g. by the presence of chlorophyll *c*_3_^7^,^10^.

Further relationships within ochrophytes were less clearly resolved and appeared to be more sensitive to the method of inference and dataset employed. Least stable was the relative position of the PX clade, the raphidophyte *Heterosigma* and the Limnista clade (Fig. 1, S6, and S7, Table 2). All three PhyloBayes analyses provided maximal support for grouping of each of these three lineages, but ML analyses provided only low support or, in the case of the SP dataset, the Limnista clade moved to a position sister to all ochrophytes. The SP dataset yielded another inconsistent result: when analysed with PhyloBayes, it recovered (with high support) the Limnista in a position sister to *Heterosigma*, disrupting the monophyly of the *Heterosigma*+PX clade. This conflicts with previous multi-gene analyses that provided strong evidence for the sisterhood of raphidophytes and the PX clade (to the exclusion of limnistan lineages)^7^,^10^. It also conflicts with groupings observed in the five remaining trees generated herein (although with only moderate or low support in two of them, Table 2).

**Figure 2:**
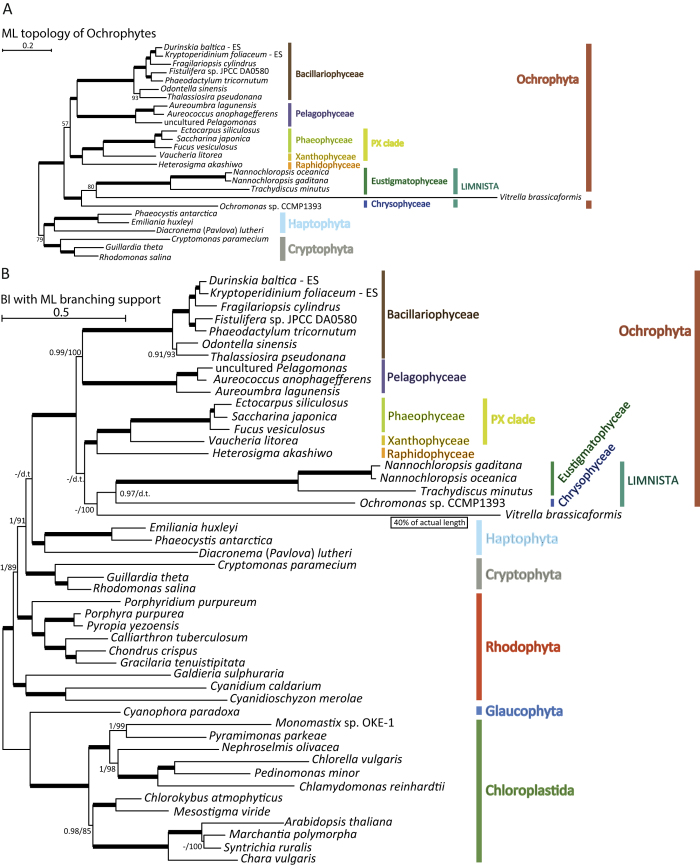
The phylogenetic position of the *Vitrella brassicaformis* plastid. The trees were inferred from a concatenated matrix of 34 slowly-evolving conserved plastid genes (dataset SG, 14,699 aa positions) excluding the rapidly evolving genome of *Karlodinium veneficum*. (**A**) Maximum-likelihood tree (RAxML, GTRGAMMA model); only the “chromalveolate” subtree is shown for simplicity. (**B**) PhyloBayes tree inferred using the CAT-GTR model. The convention for indicating branch support values is the same as in Fig. 1.

The unstable position of the Limnista clade in the different analyses may relate to significantly longer branch lengths for members of this clade compared to most other algal species (Fig. 1, 2, S6-S13), apart from the extremely long branch of the tertiary haptophyte-derived plastid of the dinoflagellate *Karlodinium veneficum*^43^. As already mentioned, long branches are known to be difficult to place reliably in phylogenetic trees, but considering that the more complex CAT-GTR model should provide more accurate inferences on the correct phylogenetic position of the rapidly-evolving limnistan branch than the site-homogeneous model^39^, we posit that branching of Limnista with the expanded PX clade is the appropriate working hypothesis.

Overall, these results point to an ochrophyte root positioned between the expanded Khakista (diatoms, pelagophytes, most likely also dictyochophytes and bolidophytes) and all other ochrophytes collectively termed the Phaeista^10^. Previous phylogenetic analyses provided conflicting and generally poorly supported results regarding the root position presumably due to the more limited phylogenetic information available at the time^5^,^6^,^8^,^10^,^42^. Notably, our inference on the position of the root of the ochrophyte phylogeny is consistent with the recently published phylogenomic analysis based on sequences of 85 nuclear genome-encoded proteins^44^. That study was not focused on ochrophytes and hence its sampling is relatively sparse, but the inferred phylogeny places the root with maximal support between diatoms and a pelagophyte on the one side and a phaeophyte and a eustigmatophyte on the other side. Our analyses suggest that further sampling of ochrophyte plastid genomes will help to completely resolve evolutionary relationships between algae within this immensely important group.

## Revisiting the phylogenetic position of plastids in “chromerid” algae

The phylogenetic analyses described above omit data from plastid-bearing alveolates (i.e., myzozoans). Plastid genomes of two prominent myzozoan subgroups – apicomplexans and dinoflagellates – are extremely divergent and have a highly reduced gene content, whereas plastids of some other myzozoans, e.g. of the oyster parasite *Perkinsus marinus* and various “colpodellids” have lost a plastid genome entirely^44^,^45^. The situation is additionally complicated by the fact that several dinoflagellate lineages have more recently acquired algal endosymbionts or plastids from other groups via a higher-order (tertiary or quaternary) endosymbiosis or kleptoplastidy^2^,^27^.

However, two recently discovered myzozoan algae, *Chromera velia*^46^ and especially *Vitrella brassicaformis*^47^, have plastid genomes that are more similar to conventional plastid genomes^12^. These two species, together hereafter called “chromerid algae” or simply “chromerids” (put in quotation marks, because they are not monophyletic within Myzozoa^44^), have several unusual characteristics that are present in dinoflagellate and apicomplexan plastids as well, supporting the notion that plastids of the different myzozoan lineages (except the dinoflagellates with plastids representing recent replacements, see above) are monophyletic. Furthermore, the lesser divergence of the plastid genome in “chromerids”, particularly *Vitrella*, allowed phylogenomic analyses of conserved plastid genes, which showed this lineage as a sister branch of ochrophyte plastids as a whole^12^. This echoes the close relationship of stramenopiles and alveolates established on the basis of nuclear genes^19^, and suggests that a plastid was present in a common ancestor of stramenopiles and alveolates, in agreement with the chromalveolate hypothesis.

Here, we used the expanded sampling of plastid genomes to reanalyze these relationships. We used the same methodology as above to analyze the F, SG and SP datasets with the addition of *Vitrella* plastid protein sequences. We also analyzed variants of all three datasets from which data from the divergent tertiary plastid of the dinoflagellate *K. veneficum* were excluded. All ML trees consistently recovered the *Vitrella* lineage nested within ochrophytes, specifically related to the Eustigmatophyceae (Fig. 2A, S8-S12). Bootstrap support (BS) values for the *Vitrella*+Eustigmatophyceae clade varied from 97% (F dataset including *K. veneficum*) to 17% (SG dataset including *K. veneficum*, Table 2). Most analyses nevertheless provided strong support for a clade comprising the Eustigmatophyceae and *Vitrella* plus *Ochromonas* (Table 2). Hence, the ML analyses very consistently showed the *Vitrella* plastid as branching with the Limnista.

Interestingly, *Vitrella* and eustigmatophytes have high similarities in their pigment composition, including violaxanthin as the dominant xanthophyll and the shared absence of chlorophyll *c* (missing also from *Chromera*), which has previously led to speculation that a specific evolutionary connection between these taxa might exist^47^. However, *Vitrella* plastid gene sequences are still quite divergent, resulting in a long branch in the inferred phylogenetic trees, longer than even that of the tertiary plastid of *K. veneficum* (Fig. 2, S10-S12). Given the inherent difficulties in placing rapidly evolving lineages in phylogenies the accuracy of these inferences are uncertain, especially since members of the Limnista clade also exhibit relatively divergent plastid genome-encoded proteins.

Indeed, using PhyloBayes and the site-heterogeneous CAT-GTR model, which should be much more effective in coping with long-branch attraction^39^, we recovered the monophyletic Limnista excluding *Vitrella* with posterior probabilities (PP) of 0.97 to 1.0, depending on the dataset (Fig. 2B, S8-S12, Table 2). In three cases the *Vitrella* plastid lineage moved to a position sister to all ochrophytes, but the monophyly of ochrophytes to the exclusion of *Vitrella* was never strongly supported (maximal support was PP of 0.84 in the F dataset including *K. veneficum*; Table 2, Figs. S8, S10, and S11). However, in the remaining three trees the *Vitrella* plastid lineage moved from the base of ochrophytes to different positions within them, either sister to diatoms and pelagophytes or sister to the Limnista clade, but support was not attained for specific branching with any ochrophyte lineage (Fig. 2B, S9, and S12, Table 2). Except for the varying position of *Vitrella*, the topology of the ochrophyte subtree in these analyses was congruent with the results obtained by analyzing the datasets without *Vitrella.*

We additionally performed an analysis of an expanded SG dataset including not only sequences from *Vitrella*, but also *Chromera* (SG+V+C dataset). The resulting trees are portrayed as Fig. S13. As expected, plastids of the two myzozoan algae branch together with maximal support in both the ML and PhyloBayes analayses. However, the branch length of *Chromera* is more than three times as long as the branch length of the already quite divergent *Vitrella* (measured from the node representing their last common ancestor). In the ML analysis the *Vitrella*+*Chromera* clade is sister to Eustigmatophyceae with BS value of 80% (Fig. S13A), consistent with the results of the ML analyses of the SG+V dataset. In the PhyloBayes analysis, the Limnista clade (to the exclusion of *Vitrella* and *Chromera*) is recovered with PP of 0.98, but the *Vitrella*+*Chromera* clade is sister to Limnista with maximal support (Fig. S13B). Given the extreme divergence of the *Chromera* plastid protein sequences evident from the analysis of the SG dataset, we did not perform analyses of the other alignment variants (F, SP).

## Did the plastid in alveolates emerge from an ochrophyte endosymbiont?

Understanding the actual nature of the relationship of plastids in ochrophytes and “chromerids” is relevant to the evolutionary history of plastids in “chromalveolates” in general. Our ML phylogenetic analyses that included *Vitrella* (or both “chromerid” algae) could suggest a tertiary endosymbiosis of a limnistan (possibly eustigmatophyte-related) alga in an alveolate host cell as the evolutionary origin of the *Vitrella* plastid. However, the clustering of the divergent “chromerid” plastid genome-encoded proteins with the relatively rapidly evolving sequences of Limnista may be a phylogenetic artifact, as suggested by the results of most analyses with the more realistic site-heterogeneous CAT-GTR model.

Some of these analyses show the *Vitrella* plastid sister to all ochrophyte plastids. This result seems consistent with the idea that *Vitrella* and ochrophytes, and by extension all alveolates and stramenopiles, share a plastid-bearing ancestor, as implied by the chromalveolate hypothesis^12^,^16^. However, the ochrophyte stem branch in all such trees is very short and poorly supported (Figs. S8, S10, and S11). This is somewhat surprising, since one would expect a relatively long and well-supported stem branch of ochrophyte plastids if the plastids of *Vitrella* and ochrophytes were vertically inherited from a common ancestor of stramenopiles and alveolates. This is because the phylogenetic distance between the last common ancestor of stramenopiles and alveolates and the last common ancestor of ochrophyte lineages seems to be rather large. Eukaryote SSU rRNA phylogenies with relaxed molecular clock models suggested that the last common ancestor of stramenopiles and alveolates may be older than the last common ancestor of ochrophytes by 250-500 million years^6^,^48^. Dating of the main diversification events in the eukaryote phylogeny based on multigene molecular clocks inferred an even higher difference in the age of these nodes – around 750 million years^49^.

Although these numbers must be interpreted with caution, it is established that the ochrophyte radiation happened hundreds of millions of years after the stramenopile stem lineage separated from the stem lineage of alveolates. Thus, under this scenario, it is unclear why monophyly of ochrophyte plastids to the exclusion of *Vitrella* is not recovered consistently. Our logic is in principle similar to that of Baurain *et al.*^23^, who pointed to the discrepancy between the strong phylogenetic signal in “chromalveolate” plastid genomes that was suggestive of common ancestry and the weak, or perhaps non-existent, phylogenetic signal in nuclear genomes for the monophyly of the “chromalveolates” as such. All these observations argue against a simple vertical inheritance of a plastid from a common ancestor of stramenopiles and alveolates, or from a hypothetical common ancestor of “chromalveolates”.

Therefore, the results of our phylogenetic analyses appear more compatible with the idea that the “chromerid” lineage gained its plastid from an early ochrophyte. The ML analyses and some PhyloBayes analyses (see Fig. 2 and S13) suggest that the donor might have been a Limnista-related alga, although these results may well result from an artefactual attraction of the very divergent protein sequences encoded by “chromerid” plastid genomes to the relatively divergent sequences of the limnistan algae. Regardless of the actual donor ochrophyte lineage, we posit that the “chromerid” plastid represents a tertiary acquisition. The derived nature of the “chromerid” plastid genomes, especially that of *Chromera*, may then be explained as a consequence of this origin by a higher-order endosymbiosis, in analogy to the highly divergent plastid genome of *K. veneficum* (note that the plastid genomes of both *Chromera* and *K. veneficum* are linear rather than circular^50^) established by a (presumably) tertiary endosymbiosis of a haptophyte in a dinoflagellate host^43^.

The actual nature of the “chromerid” plastid depends on whether the plastid in the putative ochrophyte donor is derived from a secondary endosymbiosis itself, which has been challenged^21^,^22^,^23^,^51^. Most recently, Stiller and co-workers used novel statistical analyses of the gene content in “chromalveolate” genomes to conclude that the results are incompatible with the presence of a red algal endosymbiont in an ancestor of all “chromalveolates”^25^. They instead suggested an explicit scenario, in which a secondary red-algal plastid originated in the cryptophyte lineage, from which it moved by serial endosymbioses first into an ancestor of ochrophytes and subsequently from an early ochrophyte into an ancestor of haptophytes (alveolates were not analyzed in their study). If the ochrophyte plastid really emerged from a tertiary endosymbiosis, we would modify our interpretation above such that the “chromerid” plastid is not tertiary, but quaternary, as previously speculated by Bodył and co-workers^22^. This putative endosymbiosis must have been a different event than the quaternary origin of the haptophyte plastid suggested by Stiller *et al.*.^25^, since haptophytes and alveolates never branch together in phylogenomic analyses of both plastid and nuclear genes (our results and, e.g.,^18^). Furthermore, the putative ochrophyte donor for the haptophyte plastid must have represented a very deep ochrophyte lineage preceding the radiation of extant ochrophytes, as our phylogenomic analyses leave no doubt on the monophyly of ochrophyte plastids to the exclusion of haptophyte plastids.

Previously, several lines of evidence suggested that plastids of “chromerid” algae are monophyletic with plastids of other myzozoans, i.e. apicomplexans and peridinin-containing dinoflagellates^12^. Hence, our inference of an ochrophyte origin of the “chromerid” plastid would by extension apply to the plastids in the Myzozoa as a whole. Anecdotal evidence for such a notion was actually available before, for example a phylogenetic analysis of five concatenated plastid genes carried out by Yoon *et al.* recovered peridinin-containing plastids of dinoflagellates nested within ochrophytes^52^. Our own multi-gene phylogenetic analyses that included sequences from plastid genomes of apicomplexans and peridinin-containing dinoflagellates indeed showed their grouping with “chromerids” (data not shown), but it cannot be excluded that this results is an artifact stemming from the extremely divergent nature of all these plastid genomes. Furthermore, Petersen *et al.* recently cast some doubt on the common origin of myzozoan plastids based on phylogenies of several nucleus-encoded plastid-targeted proteins^26^. While the significance of their observations remains unclear, we cautiously refrain from making definitive statements concerning all myzozoan plastids on the basis of results obtained by analyzing *Vitrella* and *Chromera* only.

## Conclusions

Our newly sequenced ochrophyte plastid genomes enabled us to improve our understanding of the ochrophyte phylogeny by providing convincing support for the existence of the Limnista clade (comprised of the classes Chrysophyceae and Eustigmatophyceae). Our studies also support positioning of the root of the ochrophyte phylogeny between the groups Khakista (diatom, bolidophytes, pelagophytes, dictyochophytes) and Phaeista (i.e. raphidophytes, phaeophytes, xanthophytes, eustigmatophytes, chrysophytes, and pinguiophytes along with a few other, smaller lineages) as redefined by Riisberg *et al.*^10^. Sampling of plastid genomes from ochrophyte classes that are as yet unsequenced and additional taxa for those like chrysophytes, for which the *Ochromonas* plastid genome sequenced herein is the sole representative, will allow testing of hypotheses on other relationships among the ochrophyte classes, such as the sisterhood of chrysophytes and synchromophytes and the probable sister relationship of the Limnista and Pinguiophyceae^7^.

The introduction of chrysophyte and additional eustigmatophyte plastid genome sequences provides a new impetus for revisiting the current ideas about the evolutionary origin of plastids in the Myzozoa. Our analyses suggest that a valid alternative hypothesis is horizontal transfer of a plastid, via endosymbiosis or kleptoplastidy, from an early ochrophyte lineage to an ancestor of “chromerid” algae. Whether this ancestor was a progenitor of the whole Myzozoa groups remains contentious due to the extremely divergent nature of all myzozoan plastid genomes characterized so far. This question may eventually be answered by additional improvements in the methodology of phylogenetic inference, new data from the nuclear genomes of *Vitrella* and *Chromera*, and/or characterization of the presently enigmatic additional plastid-bearing myzozoan lineages known by their plastid 16S rRNA genes sequences in environmental surveys^53^.

Finally, the gain of a plastid by one of the major “chromalveolate” lineages (Myzozoa) from another such lineages (Ochrophyta) suggested here does not support the chromalveolate hypothesis, but is consistent with an alternative scenario, ”the rhodoplex hypothesis”^26^. This scenario explains, for example, why plastids are not observed in non-myzozoan alveolates (ciliates, *Colponema*, and *Acavomonas*^54^,^55^): because they were never acquired by their ancestors (while the chromalveolate hypothesis requires multiple plastid losses from these lineages). The upcoming wealth of genomic data and new genome-scale comparative and phylogenetic analyses should soon enable us to critically test this newly emerging view of the evolutionary history of “chromalveolates” and their plastids.

## Additional Information

**How to cite this article**: Ševčíková, T. *et al.* Updating algal evolutionary relationships through plastid genome sequencing: did alveolate plastids emerge through endosymbiosis of an ochrophyte? *Sci. Rep.* **5**, 10134; doi: 10.1038/srep10134 (2015).

## Supplementary Material

Supplementary Information

Supplementary Tables

## Figures and Tables

**Table 1:** Basic characteristics of the newly sequenced plastid genomes.

	***T. minutus* CCALA 838**	***Ochromonas* sp. CCMP1393**
Size (bp)	120,090	126,750
Inverted repeat (bp)	9,412/ 9,411	22,910
Small single-copy region (bp)	45,210	805
Large single-copy region (bp)	56,060	80,130
Total GC content (%)	34.0	30.9
Gene content (total)	163	154
Identified protein-coding genes	129	121
Unknown or hypothetical ORFs	3	5
rRNA genes	3	3
tRNA genes	28	25

The gene counts ignore the presence of duplicated genes in inverted repeats; the split *clpC_A* and *clpC_B* genes in *Trachydiscus minutus* are counted as two separate genes.

**Table 2:**
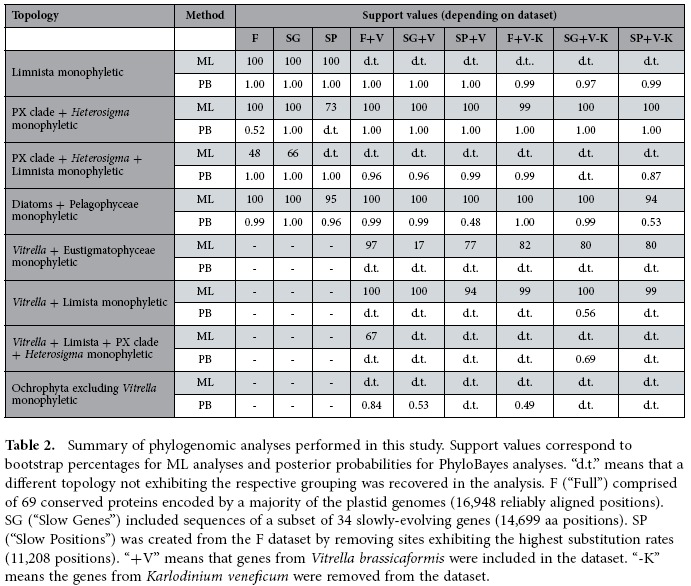
Summary of phylogenomic analyses performed in this study.
