# Supplementary material for: Updating algal evolutionary relationships through plastid genome sequencing: did alveolate plastids emerge through endosymbiosis of an ochrophyte?
Source: Sci Rep. 2015 May 28;5:10134. doi: 10.1038/srep10134 (PMC4603697; doi:10.1038/srep10134)
Supplement: Supplementary Information [file srep10134-s1.pdf]

Electronic supplementary material to:

Updating algal evolutionary relationships through plastid genome sequencing: did alveolate plastids emerge through endosymbiosis of an ochrophyte?

Tereza Ševčíková[1#], Aleš Horák[2,3#], Vladimír Klimeš[1], Veronika Zbránková[1], Elif Demir-Hilton[4], Sebastian Sudek[4], Jerry Jenkins[5], Jeremy Schmutz[6], Pavel Přibyl[7], Jan Fousek[8], Čestmír Vlček[8], B. Franz Lang[9], Miroslav Oborník[2,3], Alexandra Z. Worden[4,10*], Marek Eliáš[1*]

[1]University of Ostrava, Faculty of Science, Department of Biology and Ecology, Life Science Research Centre, Chittussiho 10, 710 00 Ostrava, Czech Republic
[2]Institute of Parasitology, Biology Centre, Czech Academy of Sciences, Branišovská 31, 370 05 České Budějovice, Czech Republic
[3]University of South Bohemia, Faculty of Science, Branišovská 1760, 370 05 České Budějovice, Czech Republic
[4]Monterey Bay Aquarium Research Institute (MBARI), Moss Landing, CA 95039, USA
[5]US Department of Energy Joint Genome Institute, Walnut Creek, California 94598, USA
[6]HudsonAlpha Institute for Biotechnology, 601 Genome Way NW, Huntsville, Alabama 35806, USA
[7]Centre for Algology and Biorefinery Research Centre of Competence, Institute of Botany, Czech Academy of Sciences, Dukelská 135, 379 82 Třeboň, Czech Republic
[8]Institute of Molecular Genetics, Czech Academy of Sciences, Vídeňská 1083, 142 20 Prague 4, Czech Republic
[9]Département de Biochimie, Centre Robert-Cedergren, Université de Montréal, 2900 Boulevard Edouard Montpetit, Montréal, Québec, H3C 3J7, Canada
[10]Integrated Microbial Biodiversity Program, Canadian Institute for Advanced Research, Toronto, M5G 1Z8, Canada

[*]Authors for Correspondence:
Marek Eliáš, Life Science Research Centre, Department of Biology and Ecology, Faculty of Science, University of Ostrava, Ostrava, Czech Republic, tel.: +420-597092329, fax: +420-597092382, e-mail: marek.elias@osu.cz
Alexandra Z. Worden, Monterey Bay Aquarium Research Institute (MBARI), Moss Landing, CA 95039, USA, e-mail: azworden@mbari.org

[#]these authors contributed equally to this work

This file includes Supplementary Methods, Supplementary Notes, and Supplementary Figures S1-S13.

Supplementary Methods

Cultivation and DNA isolation. *Trachydiscus minutus* CCALA 838 (Přibyl et al. 2012) was grown in the BBM medium (Nichols 1973) or on agar plates (2% agar in Zehnder medium; Staub 1961) at the light intensity of 40 µmol m^{-2} s^{-1} PhAR and temperature 24 ºC. *Ochromonas* sp. CCMP1393 was purchased from the NCMA (Bigelow, Maine, USA) and grown at 21ºC in the K medium (Keller et al. 1987) with reduced salinity (25% reduction) Sargasso seawater as the base and supplemented with yeast extract. The phylogenetic position of this isolate was determined by sequencing the 18S rRNA gene and performing phylogenetic analysis (Fig. S1). The culture used to attain *Ochromonas* DNA was grown from a single cell (initially sorted via flow cytometry). Total DNA was isolated using the Dellaporta protocol (Dellaporta et al. 1983) in case of *T. minutus* (using independent isolations from different cultures for different sequencing runs) and the CTAB method (Winnepenninckx et al. 1993) in case of *Ochromonas*.

DNA Sequencing and assembly. 1 µg of whole genomic DNA from *T. minutus* was subjected to 454 sequencing on the Titanium platform according to GS FLX Library Preparation Method protocols (Roche). In total over 900,00 reads were produced from a shotgun and a 3-kb paired-end library and assembled using Newbler 2.6 (Roche), resulting in ~20,000 contigs and 18.7 Mb of a unique sequence. Contigs representing the two single-copy regions and the inverted repeat region of the *T. minutus* plastid genome were identified by BLAST (Altschul et al. 1997) and manually assembled into a contiguous circular-mapping sequence by taking into account linking information from pair-end reads. To improve the assembly, Illumina reads (a PE library sequenced by GATC, Konstanz) were mapped onto the plastid genome assembly using Bowtie 2 (Langmead and Salzberg 2012); mismatches or indels were identified visually using GenomeView (Abeel et al. 2012), which led to correcting seven frame-shift errors in the original assembly and identification of a single-nucleotide indel differentiating the two otherwise identical inverted repeats. DNA from *Ochromonas* sp. CCMP1393 was sequenced using 454 and Illumina (2x100 with 4 kb average insert size). Plastid data was identified as a significant peak in the linear 454 24mer histogram at approximately 100x. Potential plastid reads were extracted if they were composed of at least 85% of the 24mers found in the vicinity of the 100x peak. An initial assembly was constructed using the combined linear 454 and Illumina paired reads using Newbler. The resulting initial assembly was composed of four contigs joined into one scaffold, which was closed into a contiguous circular-mapping sequence by re-mapping 454 reads using Blasr (Chaisson and Tesler 2012) and by mapping Illumina reads using BWA (Li and Durbin 2009). By iterative extension of the sequence into gaps and at both termini all five gaps (including the gap between the very 3'-terminus and very 5'-terminus of the original scaffold) could be closed and 19 frame-shift errors could be corrected. Mapping of the reads onto the sequence was visually checked by using GenomeView.

Annotation of the plastid genomes. Initial automated annotation of the assembled plastid genome sequences obtained using MFannot (http://megasun.bch.umontreal.ca/cgi-bin/mfannot/mfannotInterface.pl) was further checked and improved manually. Comparison to homologous sequences identified by blastp searches (Altschul et al. 1997) against the non-redundant protein sequence database at NCBI (http://blast.ncbi.nlm.nih.gov/Blast.cgi) was employed to check the assignment of gene names and selection of most likely initiation codons. All putative intergenic regions were screened by blastn and blastx searches against the non-redundant protein sequence database to identify possible short or less conserved genes. The absence of genes known from other plastid genomes was verified by tblastn searches against the *T. minutus* and *Ochromonas* sequences or by hmmer (v3.1) searches (http://hmmer.org/; Finn et al. 2011) against the genome sequences conceptually translated in all six frames with queries represented by HMM models obtained from the Pfam database (Finn et al. 2014). The identity of the *clpN* gene (see Results and discussion for details) was confirmed using the I-TASSER program (Zhang 2008) with

default settings. Genome maps (Fig. S2) were drawn using the GenomeVx program (Conant and Wolfe 2008).

Building phylogenomic matrices. Homologous proteins encoded by plastid genomes of various algae and plants were identified by BLAST (the list of plastid genomes analysed and accession numbers to sequences are provided in Table S1) and each group of homologs was analyzed by inferring a maximum-likelihood (ML) tree (sing the CpREV+F+Γ substitution model) to check for possible misidentifications, paralogs, contaminations or other sources of artifacts. 68 conserved genes we included in the analysis: acsF, atpA, atpB, atpH, atpI, ccs1, ccsA, clpC, petA,petB, petD, petG, petN, psaA, psaB, psaC, psaD, psbA, psbB, psbC, psbD, psbE, psbF, psbH, psbI, psbJ, psbK, psbN, psbT, psbV, rpl2,rpl3, rpl4, rpl5, rpl6, rpl11, rpl14, rpl16, rpl19, rpl20, rpl23, rpl27, rpl31, rpoA, rpoB, rpoC1, rpoC2, rps2, rps3, rps4, rps5, rps7, rps8, rps11, rps12, rps13, rps14, rps16, rps17, rps18, rps19, secA, secY, sufB, tatC, tufA, ycf3, ycf4. Orthologous sequences were aligned using MAFFT (Katoh et al. 2002) and unreliably aligned regions were manually discarded. Alignments were concatenated using Phyutility 2.2 (Smith and Dunn 2008), leaving 16,948 amino acid (aa) positions in the main ("Full") alignment (hereafter referred to as the dataset F). An alternative alignment was constructed to include only sequences of the slowly evolving 34 plastid genes as defined by Janouškovec et al. (2010). The respective protein sequences were aligned using MAFFT, trimmed using Gblocks (Talavera and Castresana 2007) and concatenated using FASconCAT (Kück and Meusemann 2010), yielding the dataset SG ("Slow Genes"; 14,699 aa). A topology-independent compatibility method implemented in TIGER (Cummins and McInerney 2011) was used to remove the fastest-evolving category of sites (out of 10 categories) from the full dataset F, leaving 11,208 aligned aa positions (dataset SP - "Slow Positions"). All alignments are available upon request from the corresponding author Marek Eliáš.

Phylogenetic analyses. Phylogenetic trees were inferred using two different methods. ML trees were obtained using RAxML 7.4.8a (Stamatakis 2006). The GTRGAMMA model of amino acid substitution yielded the highest likelihood score in initial analyses of concatenated datasets (with topology being consistent among all models explored), and was thus used as the only model throughout all subsequent analyses. The best scoring trees were estimated using rapid-bootstrapping from 500 replicates. The branching support was assessed using thorough ('slow') non-parametric bootstrap from 500 replicates. Bayesian inference of concatenated datasets was performed using PhyloBayes 3.3f (Lartillot et al. 2009). Cross-validation (CV) revealed that the most complex infinite mixture model (CAT), with components differing by their equilibrium frequencies, but sharing the relative exchange rates as defined by GTR model, yielded the best CV score. For each dataset, four MCMC chains were run until converged (i.e. maximum observed discrepancy was 0.3 or lower) and effective sample size of model parameters reached 100. Posterior probabilities computed after discarding first 1/5th of generations represent statistical support of branching.

Analysis of evolutionary rates of ochrophyte plastid genes. Root-to-tip distances of ochrophyte species were extracted from 51 single-gene phylogenetic trees using TreeStat (http://tree.bio.ed.ac.uk/software/treestat/). Only the plastid genes with monophyletic ochrophytes were considered and sequences from the extremely divergent plastid genomes of *V. brassicaformis* and *K. veneficum* were omitted from the phylogenetic reconstruction. The values were averaged within each of the following ochrophyte clades: diatoms, pelagophytes, extended "PX" clade (including *Heterosigma akashiwo*) and Limnista. Ratios of average root-to-tip distance comparing Limnista to each of the remaining ochrophyte clades were then calculated for every particular gene and visualized as box-plots using Prism 6 statistical package (GraphPad). Significance of deviation of distribution of ratios from 1.0 was tested using one-tailed t-test and non-parametric Wilcoxon signed-rank test as included in Prism 6.

Supplementary Notes

Supplementary Note 1 – notable features if the gene sets in the *Ochromonas* sp. CCMP1393 and *Trachydiscus minutus* plastid genomes

We found 25 and 28 different tRNA genes in the plastid genomes of *Ochromonas* sp. CCMP1393 and *Trachydiscus minutus*, respectively. The difference in the numbers of tRNA genes is due to extra tRNAs for each arginine, glycine, and leucine in the *T. minutus* plastid genome (Table S3). Both sets of tRNAs are predicted to be able to decode all existing codons if the super-wobble pairing mechanism is considered (Rogalski et al. 2008). *Ochromonas* uniquely among ochrophytes lacks the tRNA-Gly(GCC) gene and all Gly codons are thus most likely decoded by tRNA-Gly(UCC), considering super-wobble pairing between the third position of the codons and the U residue in the anticodon. *Trachydiscus* is unique among ochrophytes in having a tRNA-Leu(CAA) gene. As in bacteria and other plastids (Silva et al. 2006), the isoleucine codon AUA is apparently decoded by a tRNA [tRNA-Ile(CAU)] with the CAU anticodon post-transcriptionally modified to lysidine (2-lysyl-cytidine) at the wobble position. We could distinguish the three tRNAs with the CAU anticodon, i.e. tRNA-fMet(CAU), tRNA-Met(CAU), and tRNA-Ile (CAU), by comparison with previously annotated genes (Kurihara and Kunisawa 2004).

Additional non-coding RNAs encoded by the two plastid genomes include all three ribosomal RNAs (16S, 23S, 5S); in contrast to some other ochrophytes, genes for transfer-messenger RNA (tm-RNA) and signal recognition particle RNA (4.5S RNA encoded by the *ffs* gene) are absent (Table S2 and Fig. S2).

We evaluated variations in plastid gene content and genome architecture among eustigmatophytes on a broader phylogenetic scale than previously possible. The different *Nannochloropsis* spp. have nearly identical protein-coding gene sets (Wei et al. 2013; Starkenburg et al. 2014), whereas more pronounced differences were observed between *Nannochloropsis* and *Trachydiscus*. Specifically, *Nannochloropsis* spp. have retained the *ycf36* gene (lost in *Trachydiscus*), in contrast to *Trachydiscus*, which has retained *acpP, lysR* (=*rbcR*), and *tsf* genes (lost in *Nannochloropsis*; Table S3). Furthermore, while the *Nannochloropsis* species have little to no difference in the gene order and orientation (Wei et al. 2013; Starkenburg et al. 2014), 17 different blocks of colinear genes are apparent when the plastid genomes of *Trachydiscus* and *Nannochloropsis* spp. are compared (Fig. S2), indicating a number of rearrangements of the plastid genomes since the last common ancestor of eustigmatophytes.

Except the *ycf49* gene present in *Trachydiscus* and other eustigmatophytes, the two newly sequenced plastid genome do not harbour any genes of recognizable homology that would be absent from other ochrophytes. However, the *Ochromonas* genome includes five ORFs (putative protein-coding genes) with no discernible homology to sequences in databases, and no hints as to the possible origin of these ORFs by extreme divergence of known genes could be obtained by comparing their relative position to other genes. The *Trachydiscus* genome bears three ORFs that are most likely homologous to ORFs at equivalent positions in plastid genomes of *Nannochloropsis* spp. (Table S3); however, comparing the relative position of these ORFs to other plastid genomes provided no conclusive evidence for possible homology to known genes.

The *Ochromonas* plastid genome is unique among sequenced ochrophytes in lacking the *thiS* gene encoding a sulfur carrier protein involved in thiamine biosynthesis, whereas the *Trachydiscus* plastid genome does not seem to lack any gene that would be ubiquitous in other ochrophytes. However, sequenced eustigmatophyte plastid genomes, i.e. those of *Trachydiscus* and *Nannochloropsis* spp., share the absence of *rpl24*. This gene encodes a protein of the large ribosomal subunit and is present in all other sequenced ochrophyte plastid genomes, including

Ochromonas (Table S3), but is missing from most plastid genomes from outside ochrophytes (Maier et al. 2013). Since *Trachydiscus* and *Nannochloropsi*s represent the two main eustigmatophyte sublineages (Přibyl et al. 2012; Fawley et al. 2014), *rpl24* seems to have been lost in the eustigmatophyte stem lineage. No potential nucleus-encoded plastid-targeted homolog of the Rpl24 protein could be found in available genome sequences from *Nannochloropsi*s spp. or our transcriptome data from *Trachydiscus* (data not shown), suggesting that the eustigmatophyte plastid ribosomes could dispense with the Rpl24 protein, although it is maintained in other ochrophytes.

The genes *ycf33* and *ycf39* are poorly characterized concerning their function yet present in all ochrophyte plastids expect those of eustigmatophytes and *Ochromonas* (Table S3). If the absence of these two genes from other eustigmatophyte and chrysophyte (and synchromophyte) plastid genomes is confirmed by future sampling, these losses would constitute putative synapomorphies for the whole clade comprising these ochrophyte classes, i.e. Limnista. There are other shared, but less notable, gene absences from the eustigmatophyte and *Ochromonas* plastid genomes, specifically, genes that are missing in some other ochrophytes as well (Table S3).

Supplementary Note 2 – phylogenetic relationships outside the Ochrophyta inferred from analyses of plastid genomes

In the main text we focus on the results of our phylogenomic analyses directly relevant to the phylogeny of ochrophytes; here we briefly describe and discuss aspects of our trees (Figs 1, 2, and S6-13) concerning other algal lineages.

First, we observed an unstable position of the highly supported clade of the green algae *Mesostigma viride* and *Chlorokybus atmophyticus* (currently classified as Streptophyta) in our trees. Depending on the dataset and phylogenetic method, this clade was either a basal branch within the monophyletic Streptophyta, or was sister to all remaining Chloroplastida combined. These alternating positions were observed in previous analyses (Lemieux et al. 2007; Baurain et al. 2010; Lang and Nedelcu 2012) and the reasons for this incongruence are not clear. Regardless, the position of at the base of the Streptophyta is supported by anlayses based on nuclear and mitochondrial genes (Rodríguez-Ezpeleta et al. 2007).

Second, our analysis is the first to include full plastid genome data from the cyanidiphyte red alga *Galdieria sulphuraria* (a draft plastid genome sequence could be extracted from the data generated by the *G. sulphuraria* genome project; Schönknecht et al. 2013). *G. sulphuraria* branched together with other members of Cyanidiophyceae (*Cyanidioschyzon merolae* and *Cyanidium caldarium*) as their sister, which is in agreement with previous phylogenetic analyses based on a smaller number of markers (Yoon et al. 2006).

Third, "chromalveolate" plastids were monophyletic and consistently branched between the Cyanidiophyceae and the remaining red algae, including the Florideophyceae, Bangiophyceae, and *Porphyridium purpureum* (Porphyridiophyceae). The same general topology was recently recovered by Tajima et al. (2004). Lang and Nedelcu (2012) reported a PhyloBayes tree based on a multigene plastid dataset lacking *G. sulphuraria* and *P. purpureum* yet instead including data from unpublished plastid genome sequences from two rhodophyte lineages missing from our analysis, the Stylonematophyceae and Compsopogonophyceae. These two lineages clustered with maximal support with the Bangiophyceae and Florideophyceae to the exclusion of the Cyanidiophyceae and "chromalveolates". Collectively these results supports the insights from analyses based on datasets including much fewer genes yet better taxonomic sampling (Yoon et al. 2002) that the red algal endosymbiont giving rise to the "chromalveolate" plastids came from a deep rhodophyte lineage having diverged before the radiation of modern red algal classes except the Cyanidiophyceae.

Four, our trees differed in the relative positions of cryptophytes and haptophytes. All ML trees except one recovered them as a monophyletic group, albeit rarely with stronger support, whereas all PhyloBayes trees and the ML tree derived from the F+V dataset showed haptophytes branching with ochrophytes, although again with low support in most cases. These two alternative relationships were also observed previously in different analyses (Baurain et al. 2010; Janouškovec et al. 2010; Lang and Nedelcu 2012). The latter topology is supported by the shared unique replacement of the *rpl36* gene in the haptophyte and cryptophyte plastid genomes by horizontal gene transfer (Rice and Palmer 2006) and is in principle consistent with the existence of the hypothetical assemblage Hacrobia (or CCTH; Okamoto et al. 2009; Burki et al. 2009) having a plastid-bearing ancestor, but the most recent phylogenomic analyses based on nuclear genes (Burki et al. 2012; Yabuki et al. 2014) do not favour the existence of this assemblage. The alternative topology with the Haptophyta+Ochrophyta clade in the plastid phylogeny would be consistent with the position of the Haptophyta sister to the SAR clade (including ochrophytes) suggested by some phylogenomic analyses (Burki et al. 2012) and the ancestral presence of the plastid in the Haptophyta+SAR group; however, this grouping is not recovered in other phylogenomic analyses (e.g. Brown et al. 2013; Yabuki et al. 2014).

In summary, phylogenomic analyses are consistent with previous results in that they support a common origin of plastids in “chromalveolate” lineages, but do not provide conclusive evidence for or against another central tenet of the “chromalveolate hypothesis”, i.e. that plastids in the different “chromalveolate” lineages were inherited vertically from a hypothetical chromalveolate ancestor.

References to Supplementary Notes

Abeel T, Van Parys T, Saeys Y, Galagan J, Van de Peer Y. 2012. GenomeView: a next-generation genome browser. Nucleic Acids Res 40:e12.

Altschul SF, Madden TL, Schäffer AA, Zhang J, Zhang Z, Miller W, Lipman DJ. 1997. Gapped BLAST and PSI-BLAST: a new generation of protein database search programs. Nucleic Acids Res 25:3389-3402.

Baurain D, Brinkmann H, Petersen J, Rodríguez-Ezpeleta N, Stechmann A, Demoulin V, Roger AJ, Burger G, Lang BF, Philippe H. 2010. Phylogenomic evidence for separate acquisition of plastids in cryptophytes, haptophytes, and stramenopiles. Mol Biol Evol 27:1698-709.

Brown MW, Sharpe SC, Silberman JD, Heiss AA, Lang BF, Simpson AG, Roger AJ. 2013. Phylogenomics demonstrates that breviate flagellates are related to opisthokonts and apusomonads. Proc Biol Sci 280:20131755.

Burki F, Inagaki Y, Bråte J, Archibald JM, Keeling PJ, Cavalier-Smith T, Sakaguchi M, Hashimoto T, Horak A, Kumar S, Klaveness D, Jakobsen KS, Pawlowski J, Shalchian-Tabrizi K. 2009. Large-scale phylogenomic analyses reveal that two enigmatic protist lineages, telonemia and centroheliozoa, are related to photosynthetic chromalveolates. Genome Biol Evol 1:231-8.

Burki F, Okamoto N, Pombert JF, Keeling PJ. 2012. The evolutionary history of haptophytes and cryptophytes: phylogenomic evidence for separate origins. Proc Biol Sci 279:2246-54.

Chaisson MJ, Tesler G. 2012. Mapping single molecule sequencing reads using basic local alignment with successive refinement (BLASR): application and theory. BMC Bioinformatics 13:238.

Conant GC, Wolfe KH. 2008. GenomeVx: simple web-based creation of editable circular chromosome maps. Bioinformatics 24:861-2.

Cummins CA, McInerney JO. 2011. A method for inferring the rate of evolution of homologous characters that can potentially improve phylogenetic inference, resolve deep divergence and correct systematic biases. Syst Biol 60:833-44.

Dellaporta SL, Wood J, Hicks JB. 1983. A plant DNA minipreparation: Version II. Plant Mol Biol Rep 1:19-21.

Fawley KP, Eliáš M, Fawley MW 2014. The diversity and phylogeny of the commercially important algal class Eustigmatophyceae, including the new clade Goniochloridales. J Appl Phycol, in press.

Finn RD, Clements J, Eddy SR. 2011. HMMER web server: interactive sequence similarity searching. Nucleic Acids Res 39:W29-37.

Finn RD, Bateman A, Clements J, Coggill P, Eberhardt RY, Eddy SR, Heger A, Hetherington K, Holm L, Mistry J, Sonnhammer EL, Tate J, Punta M. 2014. Pfam: the protein families database. Nucleic Acids Res 42:D222-30.

Janouškovec J, Horák A, Oborník M, Lukeš J, Keeling PJ. 2010. A common red algal origin of the apicomplexan, dinoflagellate, and heterokont plastids. Proc Natl Acad Sci U S A 107:10949-54.

Katoh K, Misawa K, Kuma K.-i, Miyata T. 2002. MAFFT: a novel method for rapid multiple sequence alignment based on fast Fourier transform. Nucl Acids Res 30:3059-66.

Keller MD, Selvin RC, Claus W, Guillard RRL. 1987. Media for the culture of oceanic ultraphytoplankton. J Phycol 23:633-8.

Kück P, Meusemann K. 2010. FASconCAT: Convenient handling of data matrices. Mol Phylogenet Evol 56:1115-8.

Kurihara K, Kunisawa T. 2004. A gene order database of plastid genomes. Data Sci J 3:60-79.

Lang BF, Nedelcu AM. 2012. Plastid genomes of algae. In: Bock R, Knoop V eds. Genomics of chloroplasts and mitochondria. Advances in photosynthesis and respiration, volume 35. Dordrecht: Springer. pp. 59-87.

Langmead B, Salzberg SL. 2012. Fast gapped-read alignment with Bowtie 2. Nat Methods 9:357-9.

Lartillot N, Lepage T, Blanquart S. 2009. PhyloBayes 3: a Bayesian software package for phylogenetic reconstruction and molecular dating. Bioinformatics 25:2286-8.

Lemieux C, Otis C, Turmel M. 2007. A clade uniting the green algae *Mesostigma viride* and *Chlorokybus atmophyticus* represents the deepest branch of the Streptophyta in chloroplast genome-based phylogenies. BMC Biol 5:2.

Li H, Durbin R. 2009. Fast and accurate short read alignment with Burrows-Wheeler Transform. Bioinformatics 25:1754-60.

Maier UG, Zauner S, Woehle C, Bolte K, Hempel F, Allen JF, Martin WF. 2013. Massively convergent evolution for ribosomal protein gene content in plastid and mitochondrial genomes. Genome Biol Evol 5:2318-29.

Nichols HW. 1973. Growth media – freshwater. In *Handbook of phycological methods, culture methods and growth measurements* (ed. Stein J), pp. 7-24. Cambridge, Cambridge University Press.

Okamoto N, Chantangsi C, Horák A, Leander BS, Keeling PJ. 2009. Molecular phylogeny and description of the novel katablepharid *Roombia truncata* gen. et sp. nov., and establishment of the Hacrobia taxon nov. PLoS One 4:e7080.

Přibyl P, Eliáš M, Cepák V, Lukavský J, Kaštánek P. 2012. Zoosporogenesis, morphology, ultrastructure, pigment composition, and phylogenetic position of *Trachydiscus minutus* (Eustigmatophyceae, Heterokontophyta). J Phycol 48:231-42.

Rice DW, Palmer JD. 2006. An exceptional horizontal gene transfer in plastids: gene replacement by a distant bacterial paralog and evidence that haptophyte and cryptophyte plastids are sisters. BMC Biol 4:31.

Rodríguez-Ezpeleta N, Philippe H, Brinkmann H, Becker B, Melkonian M. 2007. Phylogenetic analyses of nuclear, mitochondrial, and plastid multigene data sets support the placement of *Mesostigma* in the Streptophyta. Mol Biol Evol 24:723-31.

Rogalski M, Karcher D, Bock R. 2008. Superwobbling facilitates translation with reduced tRNA sets. Nat Struct Mol Biol 15:192-8.

Schönknecht G, Chen WH, Ternes CM, Barbier GG, Shrestha RP, Stanke M, Bräutigam A, Baker

BJ, Banfield JF, Garavito RM, Carr K, Wilkerson C, Rensing SA, Gagneul D, Dickenson NE, Oesterhelt C, Lercher MJ, Weber AP. 2013. Gene transfer from bacteria and archaea facilitated evolution of an extremophilic eukaryote. Science 339:1207-10.

Silva FJ, Belda E, Talens SE. 2006. Differential annotation of tRNA genes with anticodon CAT in bacterial genomes. Nucleic Acids Res 34:6015-22.

Smith SA, Dunn CW. 2008. Phyutility: a phyloinformatics tool for trees, alignments and molecular data. Bioinformatics 24:715-6.

Stamatakis A. 2006. RAxML-VI-HPC: maximum likelihood-based phylogenetic analyses with thousands of taxa and mixed models. Bioinformatics 22:2688-90.

Starkenburg SR, Kwon KJ, Jha RK, McKay C, Jacobs M, Chertkov O, Twary S, Rocap G, Cattolico RA. 2014. A pangenomic analysis of the *Nannochloropsis* organellar genomes reveals novel genetic variations in key metabolic genes. BMC Genomics 15:212.

Staub R. 1961. Ernährungsphysiologische Untersuchungen an der planktonischen Blaualge *Oscillatoria rubescens* DC. Schweizerische Zeitschrift für Hydrologie 23:82–198.

Tajima N, Sato S, Maruyama F, Kurokawa K, Ohta H, Tabata S, Sekine K, Moriyama T, Sato N. 2014. Analysis of the complete plastid genome of the unicellular red alga *Porphyridium purpureum*. J Plant Res 127:389-97.

Talavera G, Castresana J. 2007. Improvement of phylogenies after removing divergent and ambiguously aligned blocks from protein sequence alignments. Syst Biol 56:564-77.

Wei L, Xin Y, Wang D, Jing X, Zhou Q, Su X, Jia J, Ning K, Chen F, Hu Q, Xu J. 2013. *Nannochloropsis* plastid and mitochondrial phylogenomes reveal organelle diversification mechanism and intragenus phylotyping strategy in microalgae. BMC Genomics 14:534-52.

Winnepenninckx B, Backeljau T, De Wachter R. 1993. Extraction of high molecular weight DNA from molluscs. Trends Genet 9:407.

Yabuki A, Kamikawa R, Kolisko M, Ishikawa S, Kim E, Tanabe A, Ishida K, Inagaki Y. 2014. *Palpitomonas bilix* represents a basal cryptist lineage: insight into the character evolution in Cryptista. Scie Rep 4:4641.

Yoon HS, Hackett JD, Pinto G, Bhattacharya D. 2002. The single, ancient origin of chromist plastids. Proc Natl Acad Sci U S A 99:15507-12.

Yoon HS, Müller KM, Sheath RG, Ott FD and Bhattacharya D. 2006. Defining the major lineages of red algae (Rhodophyta). J Phycol 42: 482-49.

Zhang Y. 2008. I-TASSER server for protein 3D structure prediction. BMC Bioinformatics 9:40.

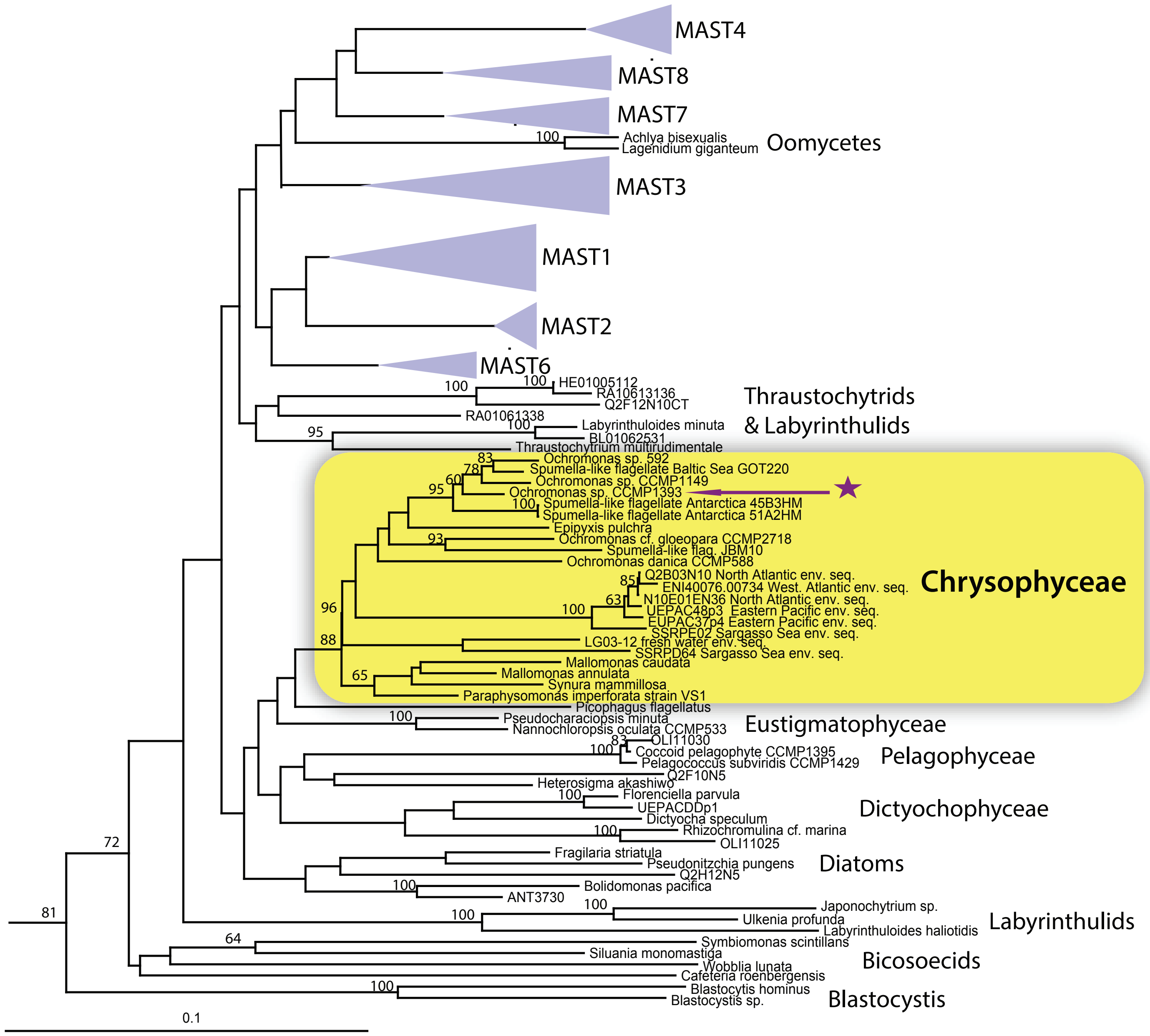

Figure S1. Phylogenetic position of *Ochromonas* sp. CCMP1393 deduced from the 18S rRNA gene sequence. Shown is a ML tree inferred using the program Phylip. *Ochromonas* sp. CCMP1393 (marked with an arrow) is nested within the class Chrysophyceae with high bootstrap support.

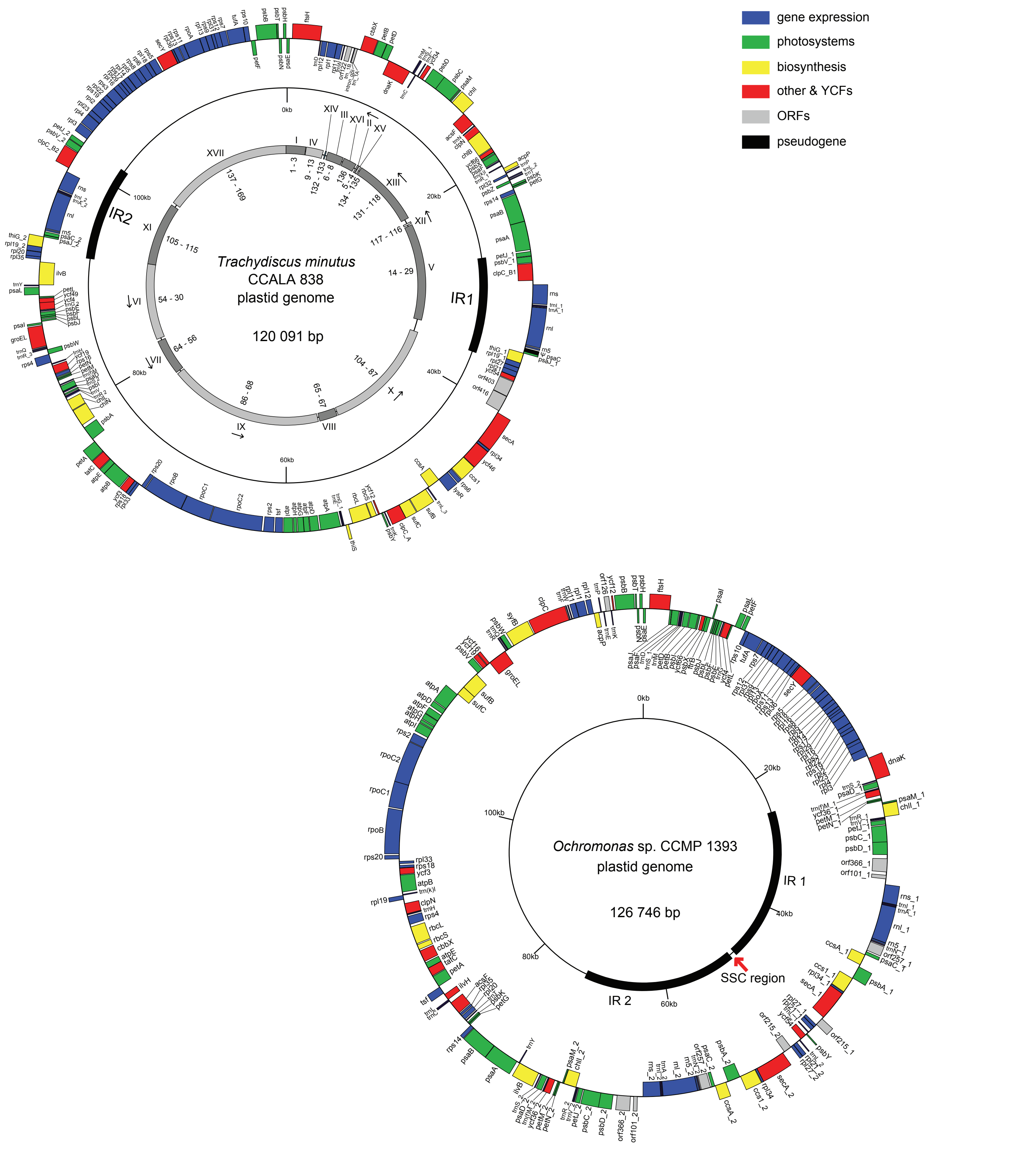

Figure S2. Genome maps of the eustigmatophyte *Trachydiscus minutus* and the chrysophyte *Ochromonas* sp. CCMP1393. Moving from the outside inwards, the rings represent genes, the extent of inverted repeats (IR1 and IR2), and (in the case of the *Trachydiscus* genome) conserved blocks of genes shared with the plastid genomes of *Nannochloropsis* spp. (the blocks are numbered by Roman numerals to reflect their relative order in the *Nannochloropsis* genomes, arrows at the Roman numerals indicate that the blocks are inverted relative to the *Trachydiscus* sequence, the Arabic numerals at the blocks indicate consecutively numbered genes in the *N. oceanica* genome that belong to the blocks). The unusually short small single copy region in the *Ochromonas* plastid genome is indicated by the red arrow. Note that genes on the outer side and inner side of the gene ring are transcribed in the clockwise orientation and counter-clockwise orientation, respectively.

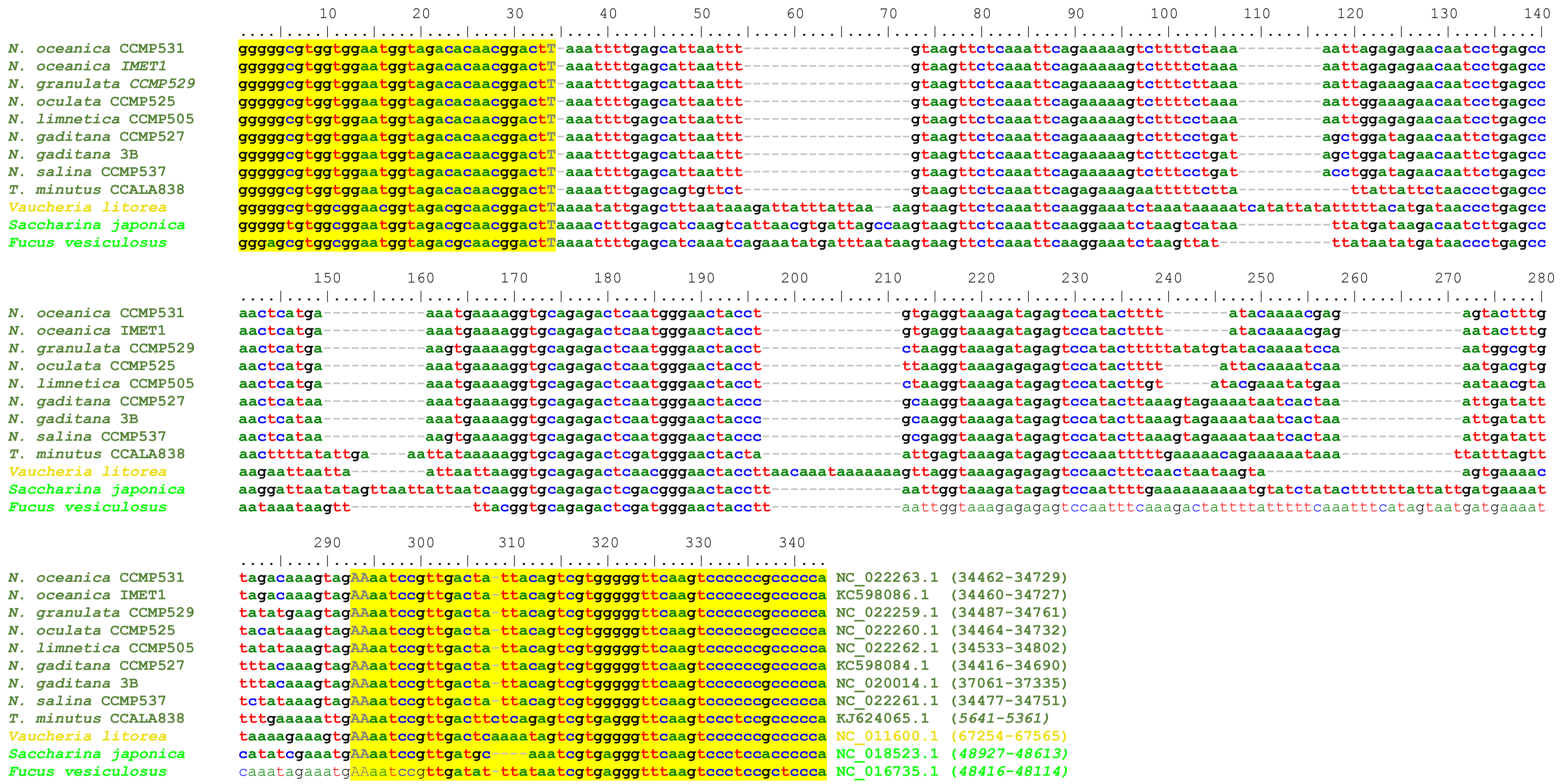

Figure S3. tRNA-Leu genes with group I introns in ochrophyte plastid genomes. The figure shows a sequence alignment of all intron-containing plastid tRNA-Leu genes from ochrophytes known to date. The actual tRNA region is highlighted in yellow; note that the intron interrupts the anticodon TAA (shown in capital letters). Species names are coloured to indicate taxomonic assignment: dark green – Eustigmatophyceae, orange – Xanthophyceae, light green – Phaeophyceae. GenBank accession numbers and coordinates of the sequence regions shown (in brackets) are provided at the end of each sequence; coordinates in italics mean that the sequence displayed is in the reverse complement orientation with respect to the database record.

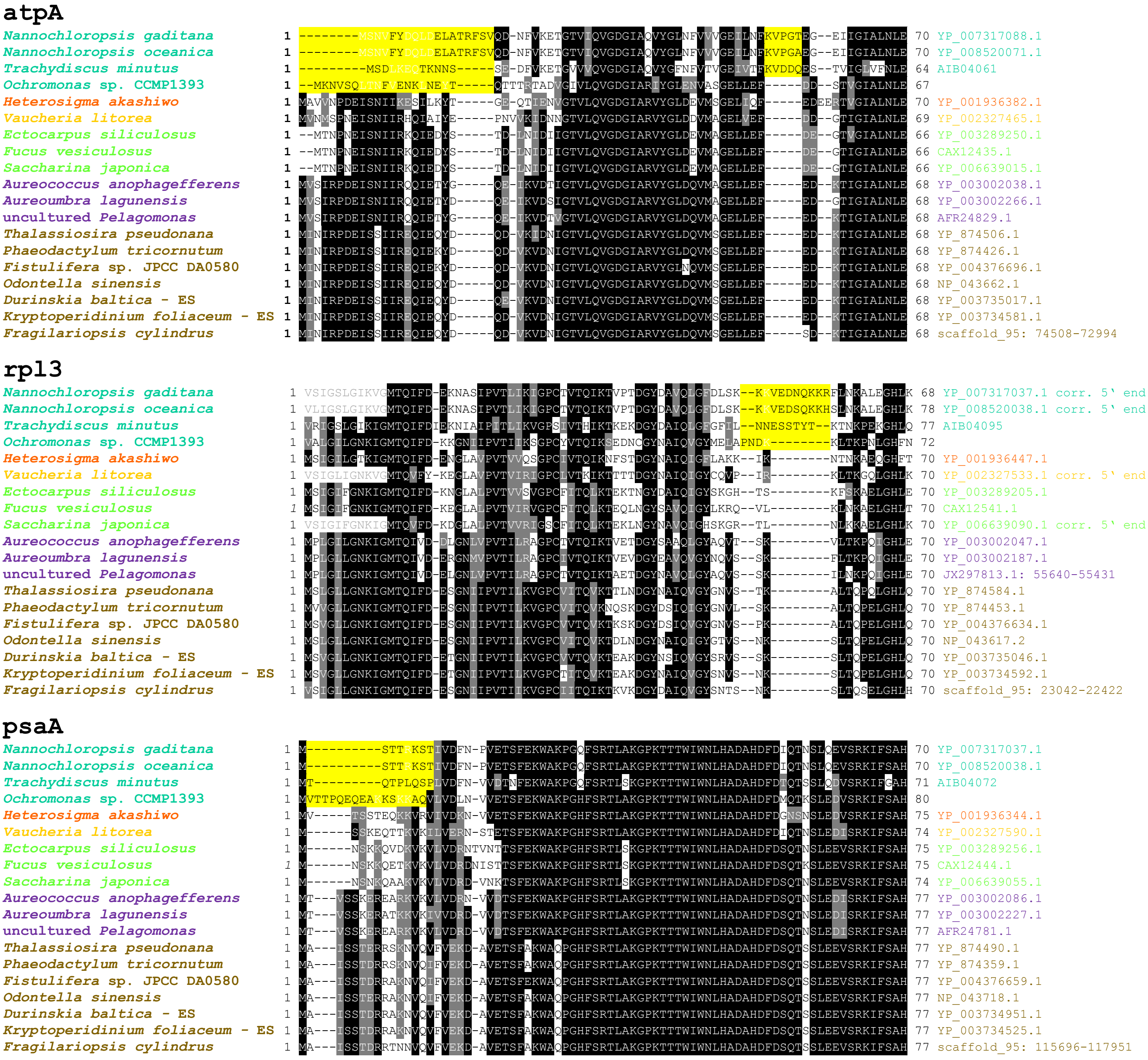

Figure S4. Examples of unusual indels in plastid-encoded proteins in the Limnista clade. Sequence alignments of plastid genome-encoded proteins from diverse ochrophytes are shown to document the increased tendency of proteins from the eustigmatophytes and chrysophytes to depart from the pattern conserved in other ochrophytes by exhibiting unusual indels (highlighted in yellow). Species names are coloured to indicate taxonomic assignment: dark green – Limnista (Eustigmatophyceae and Chrysophyceae), red – Raphidophyceae, orange – Xanthophyceae, light green – Phaeophyceae, violet – Pelagophyceae, brown – Bacillariophyceae. Sequence identifiers are provided on the right, numbers flanking the sequences indicate coordinates of the sequence regions are shown.

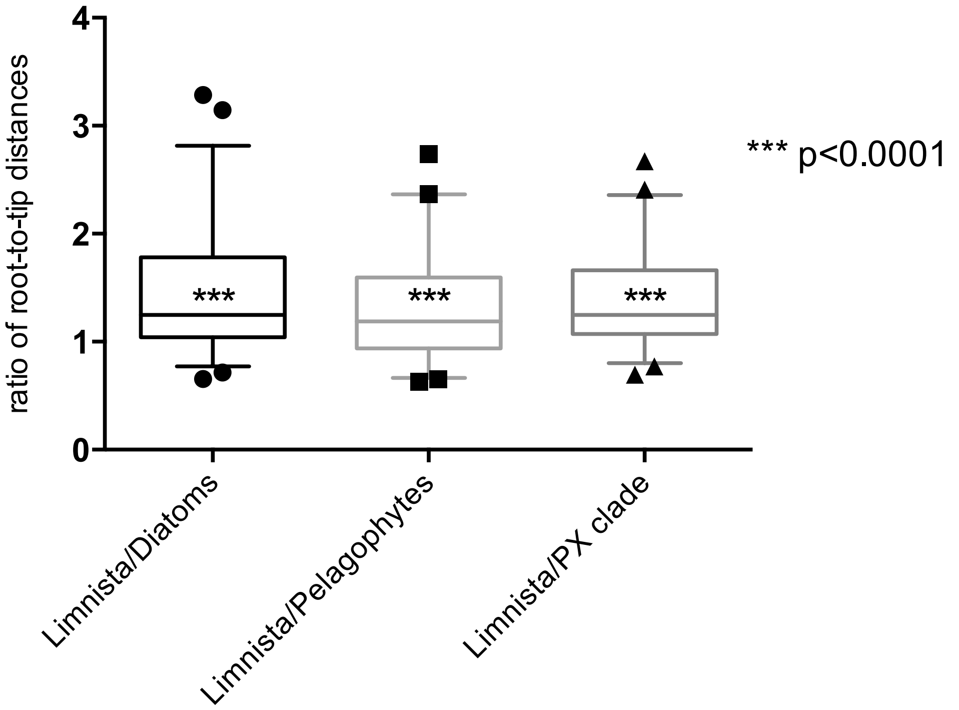

Figure S5. Rapid evolution of plastid genes of eustigmatophytes and *Ochromonas* sp. CCMP1393.
Distribution of ratios of root-to-tip distances as inferred from 51 plastid genes illustrates elevated relative rates of substitution in Limnista as compared to other ochrophyte clades.
See Supplementary Methods for details on the procedure used to obtain the data presented in the graph.

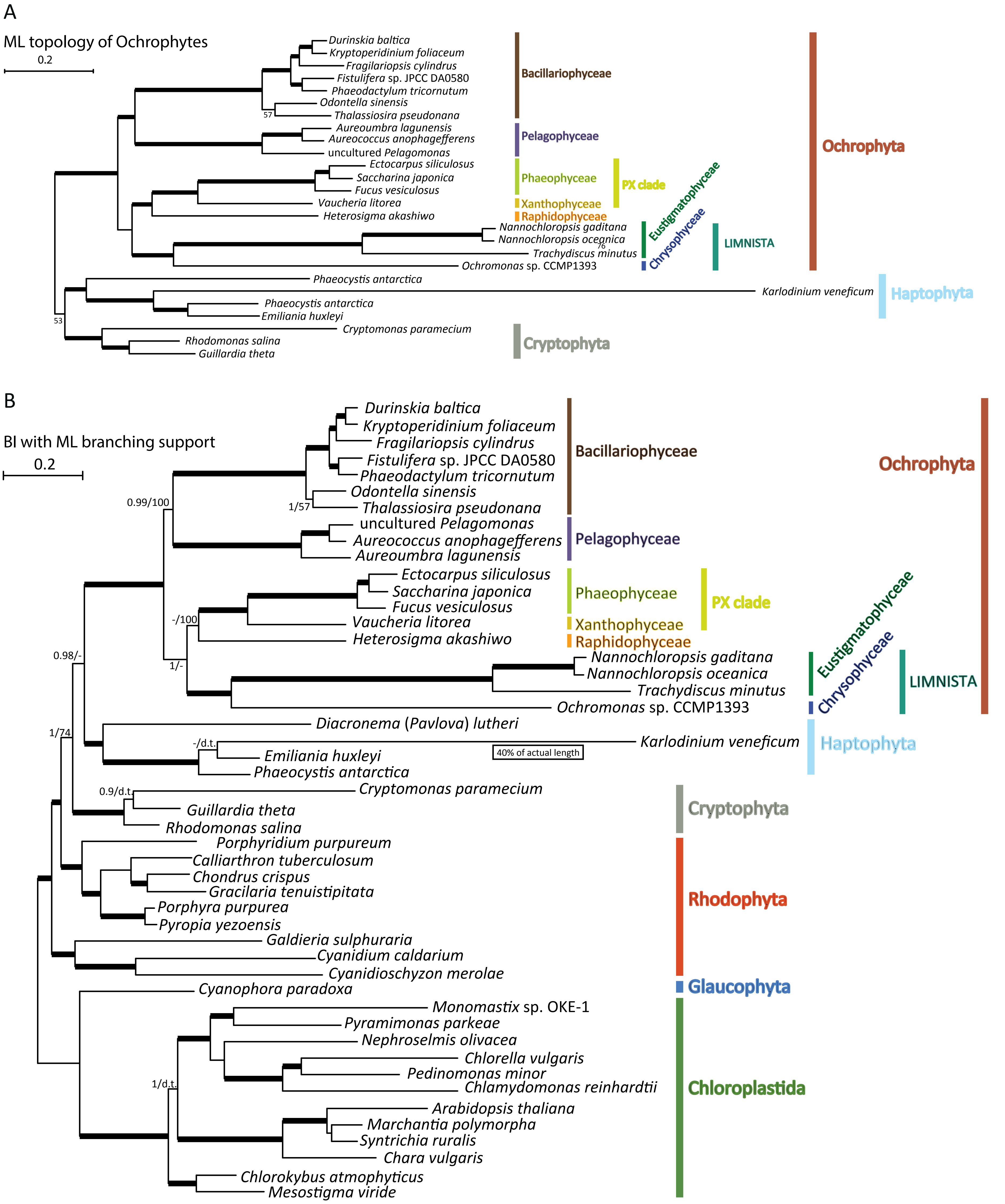

Figure S6. Plastid phylogeny inferred from protein sequences encoded by 69 conserved plastid genes (dataset F, 16,948 aa positions). (A) Maximum likelihood tree (RAxML, GTRGAMMA model); only the "chromalveolate" subtree is shown for simplicity. (B) PhyloBayes tree inferred using the CAT+GTR model. The convention for indicating branch support values is the same as in figure 1.

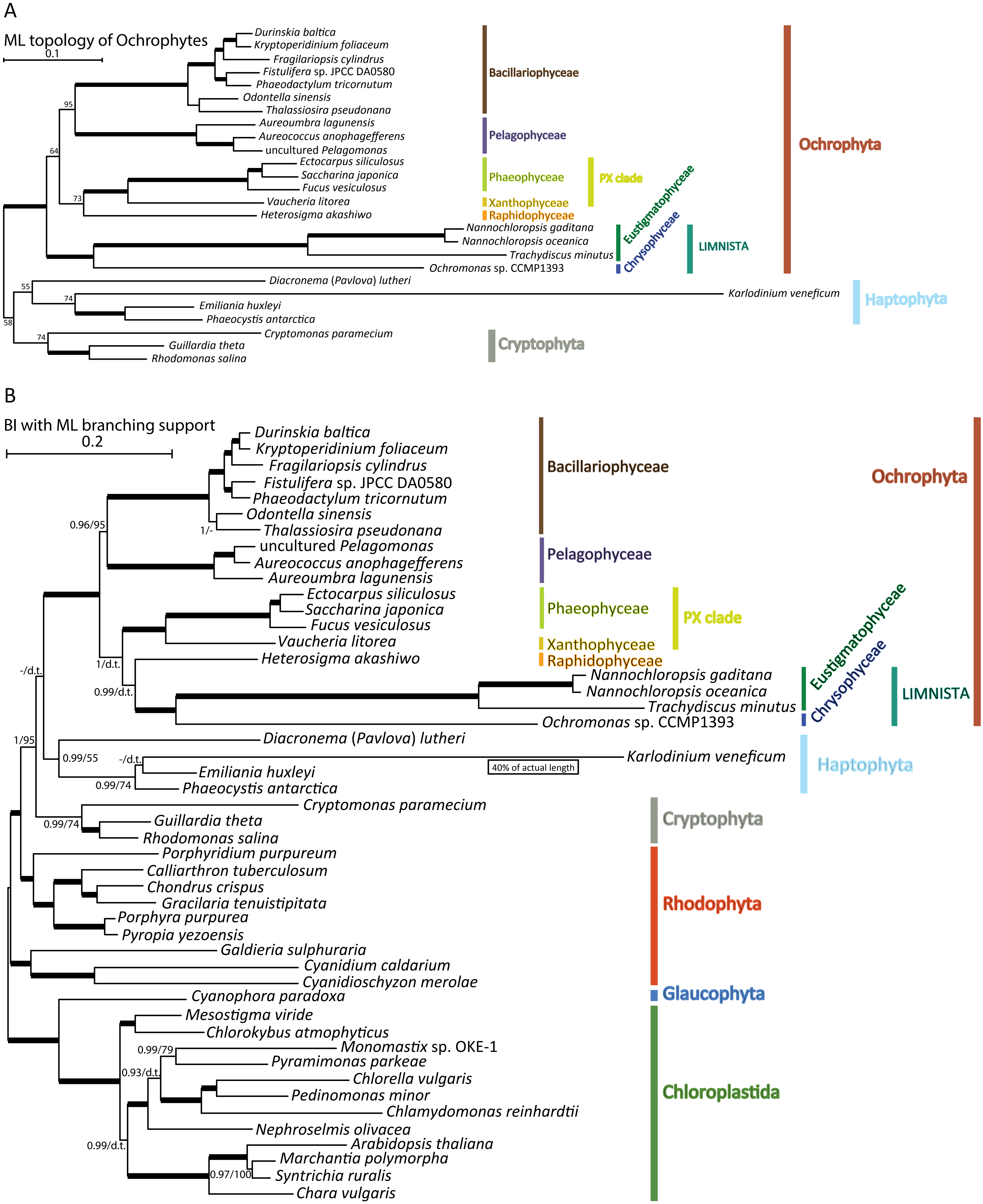

Figure S7. Plastid phylogeny inferred from protein sequences encoded by 69 conserved plastid genes after removal of the most rapidly evolving sites (dataset SP, 11,208 aa positions). (A) Maximum likelihood tree (RAxML, GTRGAMMA model); only the "chromalveolate" subtree is shown for simplicity. (B) PhyloBayes tree inferred using the CAT+GTR model. The convention for indicating branch support values is the same as in figure 1.

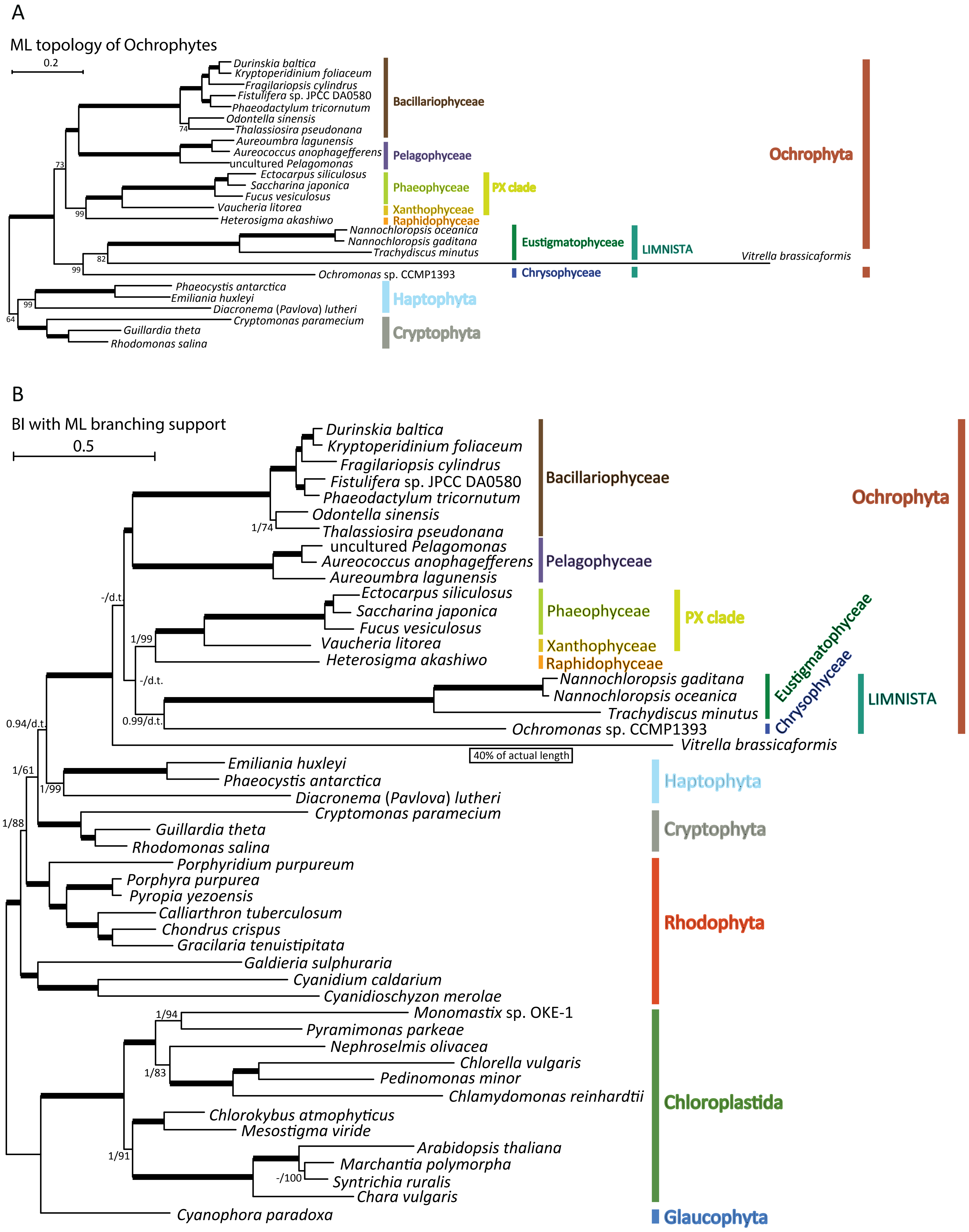

Figure S8. Plastid phylogeny inferred from protein sequences encoded by 69 conserved plastid genes, including genes from *Vitrella brassicaformis* but not from *Karlodinium veneficum* (dataset F+V-K, 16,948 aa positions).
(A) Maximum likelihood tree (RAxML, GTRGAMMA model); only the "chromalveolate" subtree is shown for simplicity.
(B) PhyloBayes tree inferred using the CAT+GTR model. The convention for indicating branch support values is the same as in figure 1.

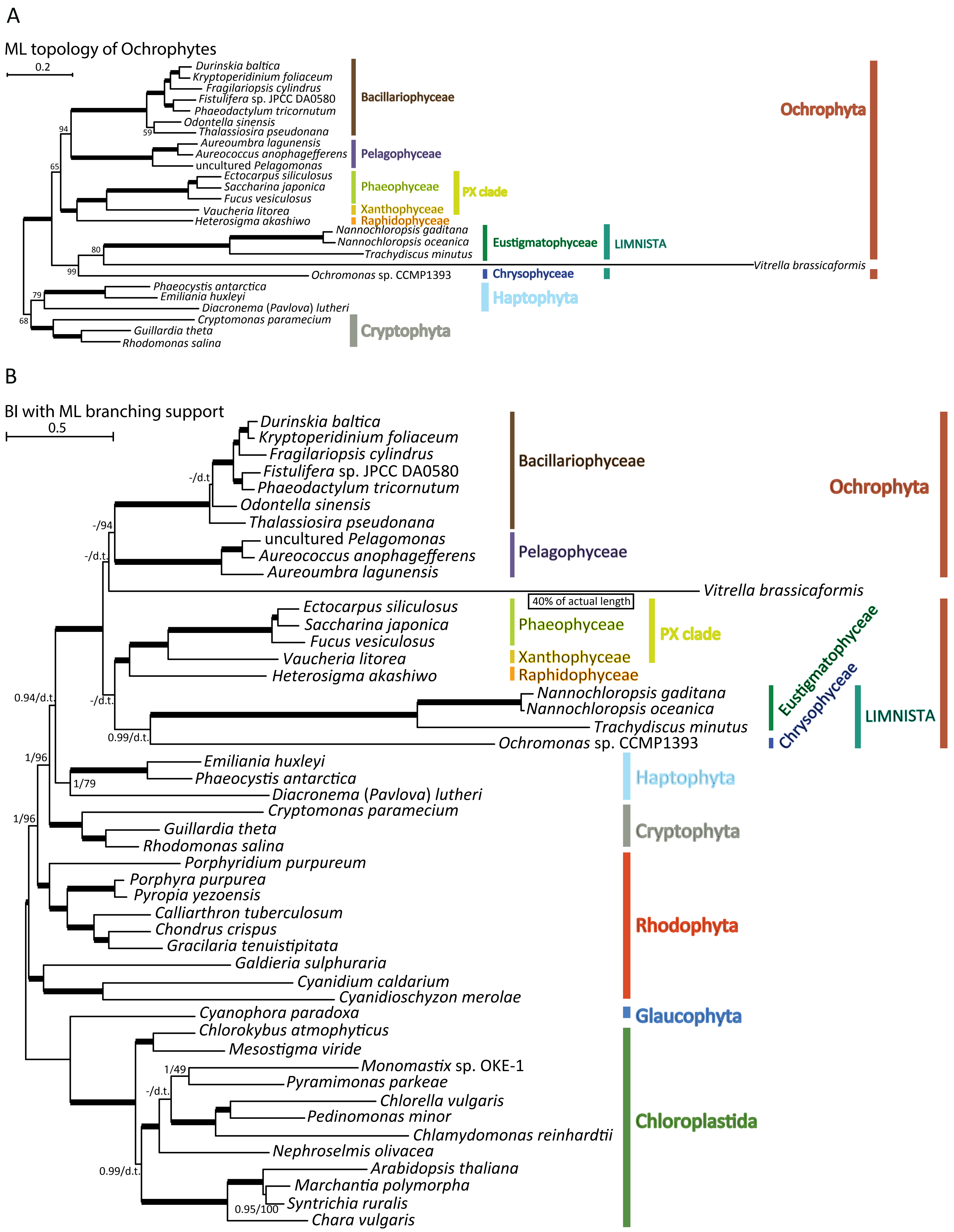

Figure S9. Plastid phylogeny inferred from protein sequences encoded by 69 conserved plastid genes after removal of the most rapidly evolving sites, including genes from *Vitrella brassicaformis* but not from *Karlodinium veneficum* (dataset SP+V-K, 11,208 aa positions). (A) Maximum likelihood tree (RAxML, GTRGAMMA model); only the "chromalveolate" subtree is shown for simplicity. (B) PhyloBayes tree inferred using the CAT+GTR model. The convention for indicating branch support values is the same as in figure 1.

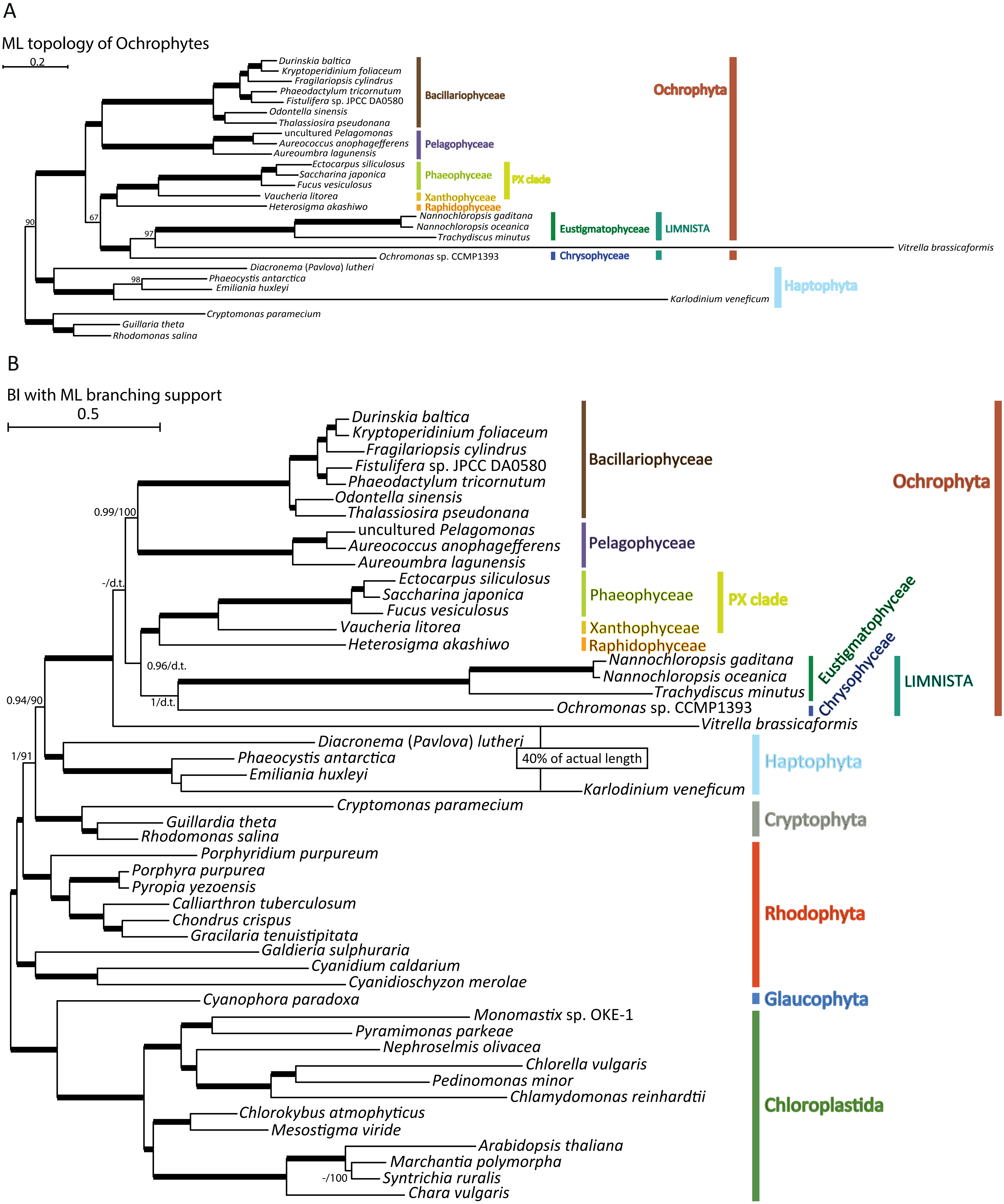

Figure S10. Plastid phylogeny inferred from protein sequences encoded by 69 conserved plastid genes, including genes from both *Vitrella brassicaformis* and *Karlodinium veneficum* (dataset F+V, 16,948 aa positions).
(A) Maximum likelihood tree (RAxML, GTRGAMMA model); only the "chromalveolate" subtree is shown for simplicity.
(B) PhyloBayes tree inferred using the CAT+GTR model. The convention for indicating branch support values is the same as in figure 1.

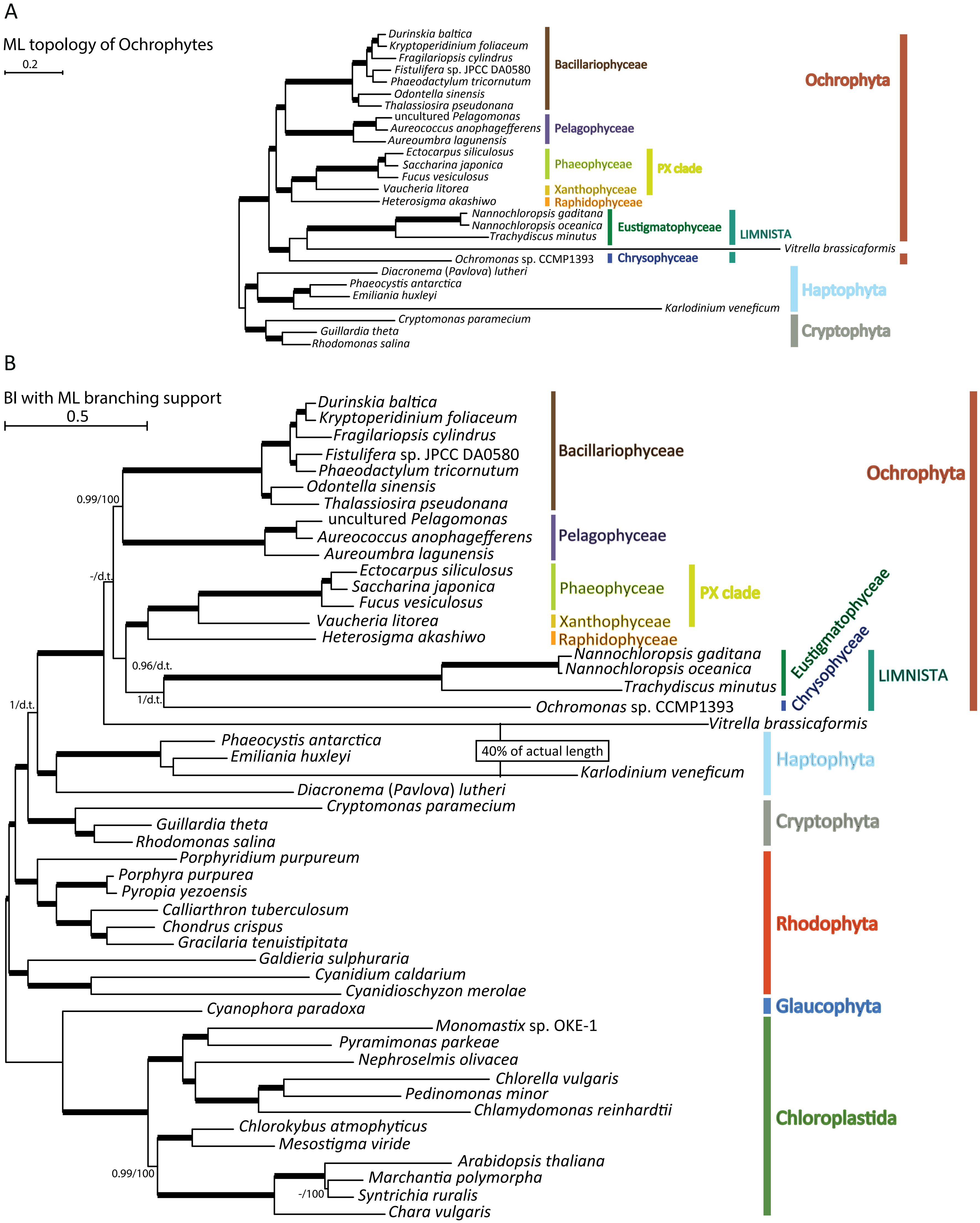

Figure S11. Plastid phylogeny inferred from protein sequences encoded by 34 slowly-evolving conserved plastid genes, including genes from both *Vitrella brassicaformis* and *Karlodinium veneficum* (dataset SG+V 14,699 aa positions).
(A) Maximum likelihood tree (RAxML, GTRGAMMA model); only the "chromalveolate" subtree is shown for simplicity.
(B) PhyloBayes tree inferred using the CAT+GTR model. The convention for indicating branch support values is the same as in figure 1.

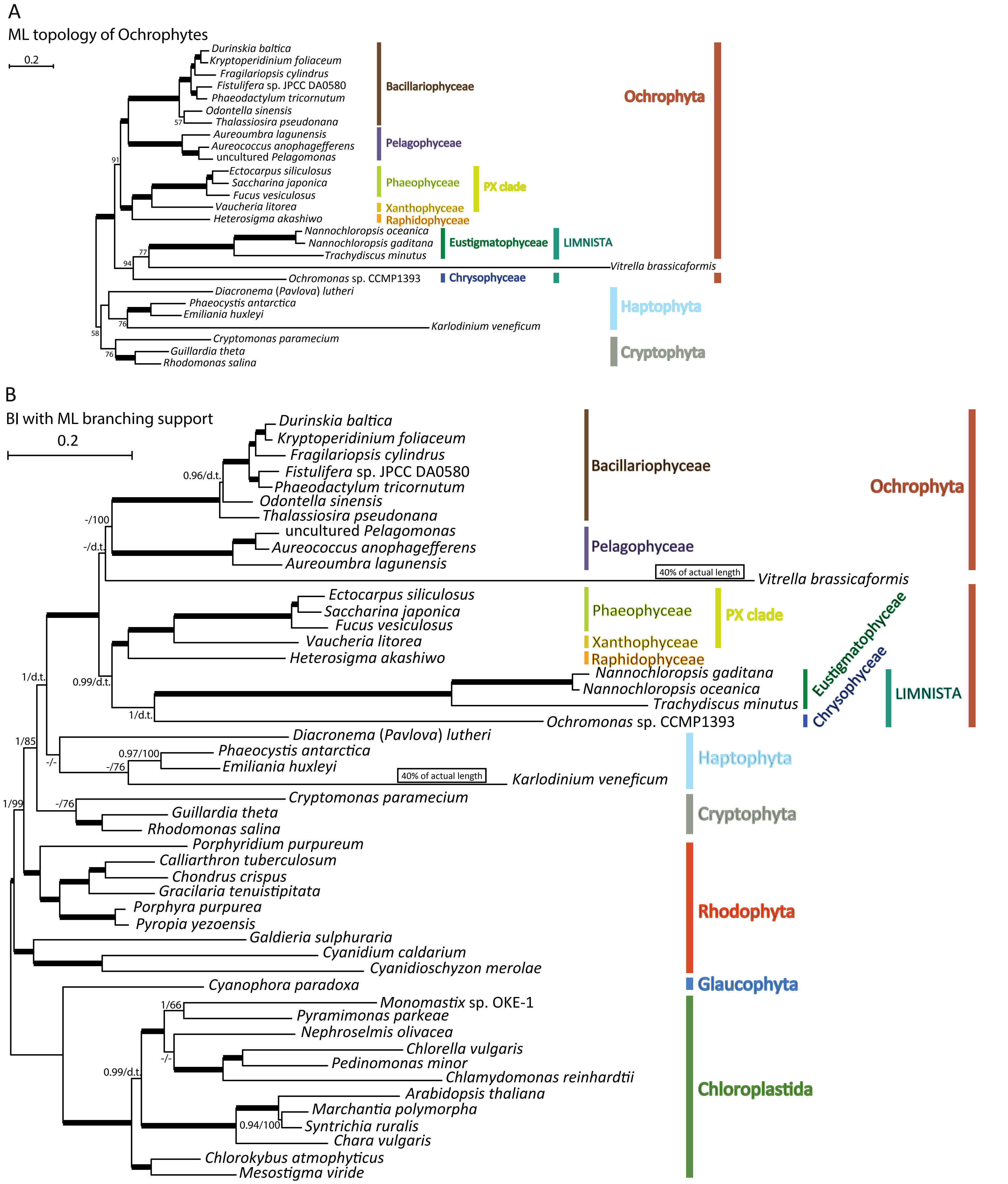

Figure S12. Plastid phylogeny inferred from protein sequences encoded by 69 conserved plastid genes after removal of the most rapidly evolving sites, including genes from both *Vitrella brassicaformis* and *Karlodinium veneficum* (dataset SP+V 11,208 aa positions).
(A) Maximum likelihood tree (RAxML, GTRGAMMA model); only the "chromalveolate" subtree is shown for simplicity.
(B) PhyloBayes tree inferred using the CAT+GTR model. The convention for indicating branch support values is the same as in figure 1.

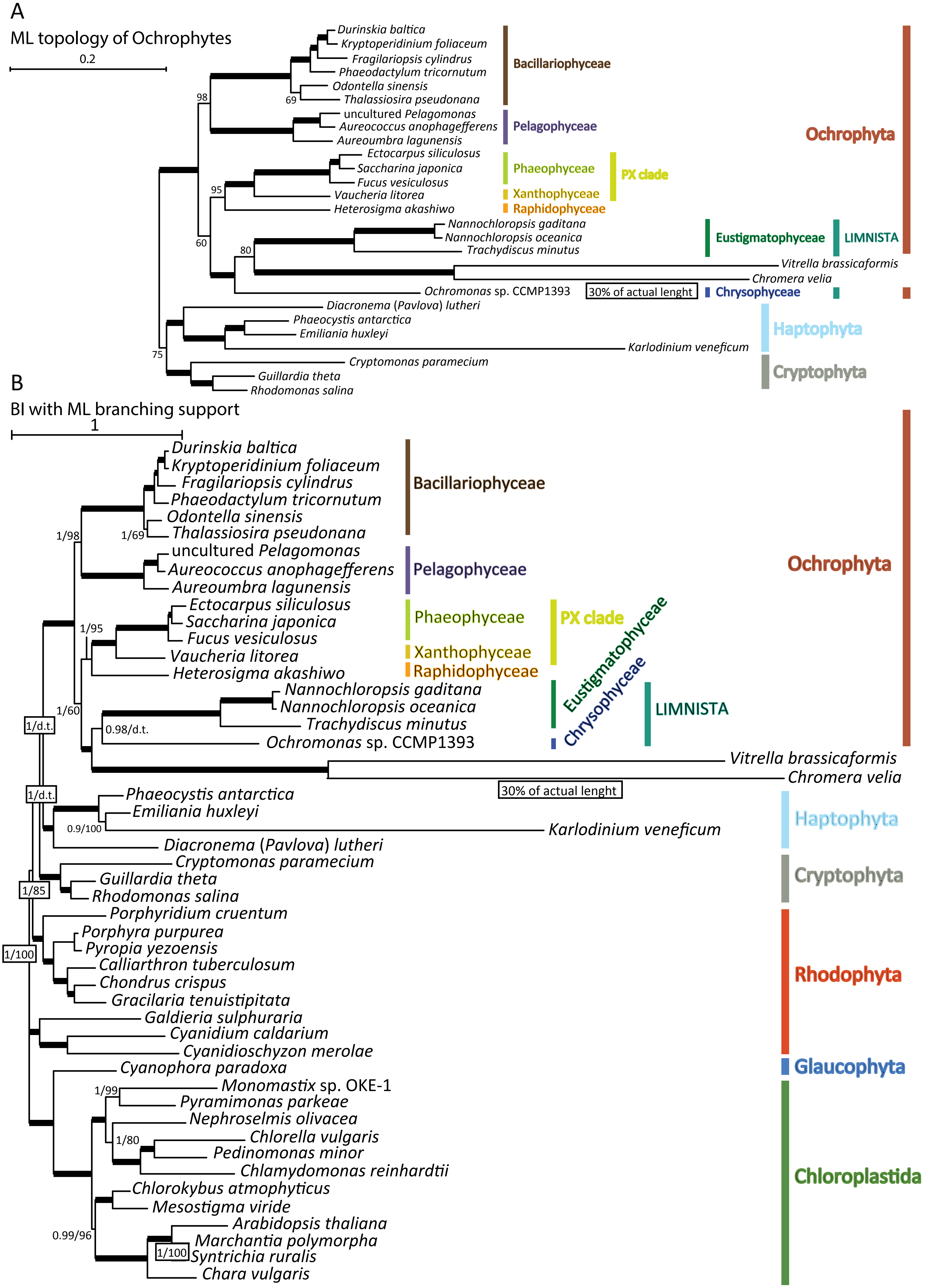

Figure S13. Plastid phylogeny inferred from protein sequences encoded by 34 slowly-evolving conserved plastid genes, including genes from *Vitrella brassicaformis, Chromera velia* and *Karlodinium veneficum* (dataset SG+V 14,699 aa positions).
(A) Maximum likelihood tree (RAxML, GTRGAMMA model); only the “chromalveolate” subtree is shown for simplicity.
(B) PhyloBayes tree inferred using the CAT+GTR model. The convention for indicating branch support values is the same as in figure 1.
